# HIV-driven tumor ecosystem: from chronic immune dysfunction to cancer evolution

**DOI:** 10.3389/fmicb.2026.1901762

**Published:** 2026-09-02

**Authors:** Guangchao Zhou, Juanyan Liao, Haolin Tang, Xingyu Hou, Qing Li, Xia Li

**Affiliations:** 1 Department of General Medicine, West China Hospital, Sichuan University, Chengdu, Sichuan, China; 2 Department of Biotherapy, Cancer Center, West China Hospital, Sichuan University, Chengdu, Sichuan, China; 3 West China School of Medicine, West China Hospital, Sichuan University, Chengdu, Sichuan, China

**Keywords:** antiretroviral therapy, cancer evolution, chronic immune dysfunction, HIV, immune evasion, immunotherapy, tumor ecosystem

## Abstract

Although HIV-associated malignancies have been reviewed separately through the lens of individual oncogenic viruses (KSHV, EBV, HPV) or immune dysfunction in isolation, no prior synthesis has integrated these processes into a single, systemic “tumor ecosystem” framework spanning virology, immunology, the tumor microenvironment, and clinical treatment response. Human immunodeficiency virus (HIV) infection profoundly alters the immune landscape, establishing a distinctive tumor ecosystem characterized by chronic immune dysfunction, inflammatory microenvironment remodeling, and virus-driven oncogenic mechanisms that collectively promote cancer initiation and progression. Despite advances in antiretroviral therapy (ART), HIV-infected individuals remain at increased risk for various malignancies, underscoring the complex interplay between persistent immune impairment and tumor evolution. This review comprehensively examines the components and dynamic changes within the tumor ecosystem of HIV-infected patients, focusing on how HIV-mediated CD4+ T-cell depletion, immune checkpoint dysregulation, chronic antigenic stimulation, and direct effects of viral proteins such as Tat and Nef facilitate tumor immune evasion and genomic instability. Integrating insights from cutting-edge single-cell omics, spatial transcriptomics, and animal model studies, we delineate the evolutionary trajectories of diverse HIV-associated cancers, including lymphomas and Kaposi's sarcoma. Furthermore, we critically evaluate the impact of ART on modulating the tumor microenvironment and immune responses, alongside emerging immunotherapeutic approaches such as immune checkpoint inhibitors and chimeric antigen receptor T-cell (CAR-T) therapies. By systematically synthesizing molecular mechanisms, clinical manifestations, and therapeutic advancements, this review aims to provide a comprehensive theoretical framework to guide precision prevention and treatment strategies for HIV-related malignancies. By integrating virological, immunological, and single-cell/spatial evidence into an ecosystem-level model, this review addresses this gap and offers direct implications for immunotherapy stratification and precision management of HIV-associated malignancies.

## 1 Introduction

HIV infection remains a global health challenge, not only due to its direct immunosuppressive effects but also because of its complex interplay with oncogenesis. The advent and widespread use of combination antiretroviral therapy (cART) have significantly improved the survival of people living with HIV (PLWH), transforming HIV infection into a chronic manageable condition. However, this increased longevity has been accompanied by a shift in the cancer landscape among PLWH, characterized by a decline in AIDS-defining cancers (ADCs) and a concomitant rise in non-AIDS-defining cancers (NADCs). This epidemiological transition underscores the need to understand the unique tumor ecosystem driven by HIV, where chronic immune dysfunction, persistent inflammation, and direct viral oncogenic mechanisms converge to influence cancer development and progression.

The conventional tumor microenvironment (TME) framework centers on the local tumor, its immediate stroma, and infiltrating immune cells at the tissue level. The “tumor ecosystem” concept used throughout this review extends beyond the local TME to incorporate systemic determinants unique to HIV infection—chronic systemic immune activation, gut-microbial translocation, ART-modified metabolic and inflammatory states, and pre-existing oncogenic viral reservoirs (KSHV, EBV)—that shape tumor initiation and evolution prior to, and independently of, local tissue architecture. This distinction motivates a systemic, longitudinal framework for HIV-associated tumors rather than a purely local TME description.

Epidemiological studies across diverse geographic regions consistently demonstrate that PLWH bear a disproportionately higher burden of both ADCs and NADCs compared to the general population. For instance, nationwide longitudinal surveillance in Japan revealed an increasing incidence of NADCs over a 23-year period, with lung, colorectal, gastric, and liver cancers being the most prevalent (Tanaka et al., 2024). Similarly, population-based cohorts in South Korea and Brazil have reported elevated standardized incidence ratios (SIRs) for ADCs such as Kaposi sarcoma, non-Hodgkin lymphoma (NHL), and cervical cancer, alongside NADCs including anal, lung, liver, and oropharyngeal cancers. These findings are echoed in sub-Saharan Africa, where HIV-HBV co-infection exacerbates hepatocellular carcinoma (HCC) risk and worsens prognosis. The increased cancer risk in PLWH is multifactorial, involving immunodeficiency, co-infections with oncogenic viruses, lifestyle factors, and the direct effects of HIV proteins on host cellular pathways.

Central to the HIV-driven tumor ecosystem is the chronic immune dysfunction characterized by persistent immune activation and exhaustion. HIV infection leads to sustained depletion and dysfunction of CD4+ T cells, impaired cytotoxic T-lymphocyte responses, and dysregulated cytokine networks, which collectively compromise immune surveillance against emerging malignant clones. Notably, low CD4/CD8 ratios and elevated CD8+ T-cell counts have been associated with increased risks of Kaposi sarcoma and NHL, respectively, even in the context of effective cART and viral suppression (Caby et al., 2021). This immune dysregulation facilitates the persistence and reactivation of oncogenic viruses such as Kaposi sarcoma-associated herpesvirus (KSHV), Epstein-Barr virus (EBV), human papillomavirus (HPV), and hepatitis B and C viruses (HBV, HCV), which are implicated in the pathogenesis of many ADCs and NADCs.

Beyond immune impairment, HIV exerts direct oncogenic effects through viral proteins such as Tat, Nef, and Vpr, which modulate host cell signaling pathways, promote genomic instability, and enhance angiogenesis (Obisesan et al., 2021). These viral factors can induce oxidative stress, mitochondrial dysfunction, and epigenetic alterations that create a pro-tumorigenic microenvironment. For example, studies have demonstrated concordant elevations of cancer-associated cytokines and mitochondrial DNA deletions in PLWH and HCC patients, highlighting shared molecular mechanisms driving carcinogenesis (Aboagye et al., 2025). Moreover, recent advances in single-cell and spatial transcriptomics have begun to unravel the complex cellular heterogeneity and intercellular communication networks within the tumor microenvironment of HIV-associated cancers, revealing immune cell reprogramming and clonal evolution dynamics that underpin tumor progression and therapeutic resistance.

The clinical implications of HIV-driven tumor ecology are profound. PLWH with cancer often present with more advanced disease, exhibit faster tumor progression, and experience poorer survival outcomes compared to HIV-negative counterparts. For instance, HIV infection is associated with earlier onset and worse prognosis of HCC, despite virologic suppression and adherence to antiretroviral therapy. Similarly, survival disparities have been reported in Kaposi sarcoma and NHL, although access to comprehensive cancer treatment and supportive care can mitigate these differences. Importantly, studies indicate that HIV status alone should not preclude standard cancer therapies, including surgery, chemotherapy, radiotherapy, and immunotherapy, as PLWH can achieve comparable treatment responses when managed appropriately.

The evolving epidemiology of HIV-associated malignancies necessitates integrated approaches to cancer prevention, screening, and management tailored to the unique needs of PLWH. Early initiation and adherence to cART remain critical for reducing the incidence of ADCs and certain virus-related NADCs by restoring immune competence and suppressing HIV replication. Concurrently, lifestyle modifications, vaccination against oncogenic viruses, and routine cancer screening are essential components of comprehensive care. Emerging biomarkers, such as cell-free DNA methylation-based inflammation scores, offer promising avenues for early detection of cancers like HCC in high-risk HIV populations (Kim et al., 2025). Furthermore, the integration of novel immunotherapeutic strategies, including immune checkpoint inhibitors, holds potential to improve outcomes in HIV-associated cancers, although the inclusion of PLWH in clinical trials remains limited.

**Figure 1:**
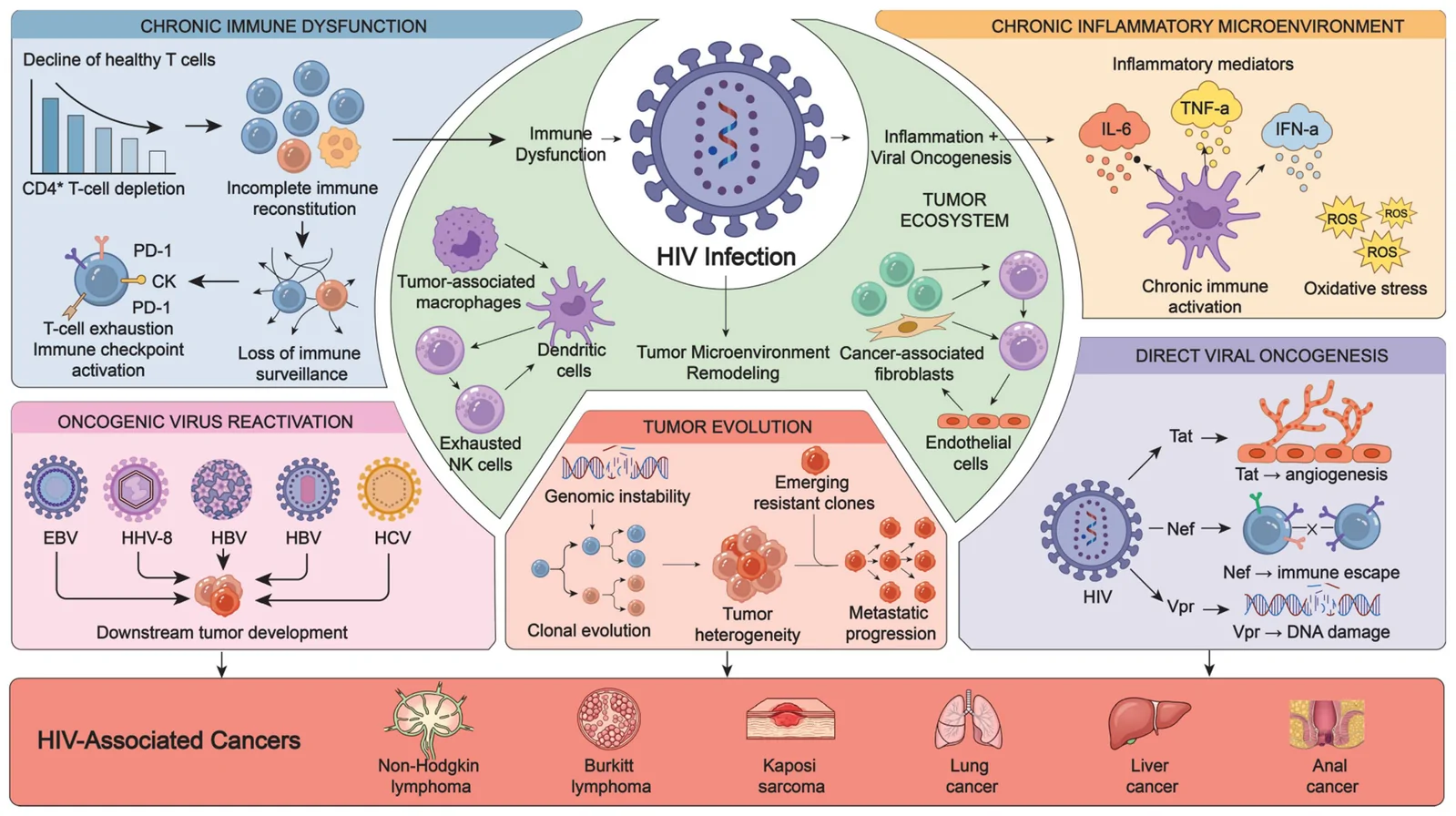
HIV-driven tumor ecosystem: from chronic immune dysfunction to cancer evolution. HIV infection establishes a unique tumor ecosystem characterized by persistent immune dysfunction, chronic inflammation, viral oncogenesis, and impaired immune surveillance. HIV-mediated CD4+ T-cell depletion, chronic immune activation, and immune checkpoint dysregulation promote immune exhaustion and facilitate the persistence of oncogenic viruses, including EBV, human herpesvirus 8 (HHV-8), HPV, hepatitis B virus (HBV), and hepatitis C virus (HCV). Concurrently, viral proteins such as Tat, Nef, and Vpr induce inflammation, immune evasion, angiogenesis, and genomic instability, collectively driving tumor initiation, clonal evolution, and progression toward both AIDS-defining and non-AIDS-defining malignancies.

In summary, HIV infection orchestrates a distinct tumor ecosystem characterized by chronic immune dysfunction, persistent inflammation, and direct viral oncogenesis, which collectively drive the increased incidence and aggressive behavior of both AIDS-defining and non-AIDS-defining cancers. Understanding the complex interplay of these factors is essential for developing effective prevention, diagnostic, and therapeutic strategies to improve cancer outcomes in PLWH. This review will systematically explore the composition of the HIV-driven tumor ecosystem, the mechanisms of immune dysfunction and viral oncogenesis, tumor evolutionary pathways, current treatment modalities, and future research directions to advance the field of HIV-associated oncology (Figure 1).

## 2 HIV infection and chronic immune dysfunction

### 2.1 CD4+ T-cell depletion and incomplete immune reconstitution

HIV infection is characterized by a progressive depletion of CD4+ T lymphocytes, a hallmark that underpins the immunodeficiency observed in affected individuals. Even with the widespread use of ART, which effectively suppresses viral replication, a subset of patients fails to achieve full immune reconstitution, resulting in persistently low CD4+ T-cell counts below normal levels. This incomplete immune recovery, often termed immunological non-response or poor immune reconstitution, is associated with ongoing immune dysfunction and increased susceptibility to opportunistic infections and malignancies. The mechanisms underlying CD4+ T-cell depletion and impaired recovery are multifactorial, involving both quantitative loss and qualitative dysfunction of these cells.

Functionally exhausted CD4+ T cells exhibit diminished proliferative capacity and reduced secretion of critical cytokines such as interleukin-2 (IL-2) and interferon-gamma (IFN-γ), which are essential for orchestrating effective immune responses. This functional impairment extends to their helper roles, including support for B cells in antibody production and for CD8+ cytotoxic T lymphocytes in antiviral activity. Studies have demonstrated that the dysregulation of T follicular helper (Tfh) cells, a specialized subset of CD4+ T cells that facilitate germinal center reactions and memory B-cell function, correlates with poor immune recovery during ART. Specifically, alterations in Tfh cell subsets and memory B-cell dysfunction at early stages of ART are predictive of suboptimal CD4+ T-cell restoration, suggesting that the interplay between these cell types is critical for immune reconstitution (Liu et al., 2023).

The clinical consequences of incomplete immune reconstitution are profound. Patients with persistently low CD4+ T-cell counts despite viral suppression remain at elevated risk for AIDS-defining cancers such as non-Hodgkin lymphoma and Kaposi's sarcoma. This is attributable to the central role of CD4+ T cells in antiviral and antitumor immunity, where their depletion compromises immune surveillance and control of oncogenic viruses like Epstein-Barr virus and human herpesvirus 8. Moreover, the immune activation and chronic inflammation associated with HIV infection further exacerbate immune dysfunction, contributing to the pathogenesis of malignancies and other comorbidities (Lutze et al., 2020).

Gender differences have also been observed in the dynamics of CD4+ T-cell decline and immune recovery. Females tend to experience a more rapid decline in CD4+ T-cell counts and faster HIV disease progression compared to males, although the underlying biological mechanisms remain to be fully elucidated. This disparity underscores the need for sex-specific considerations in monitoring and managing HIV infection (Parsa et al., 2020).

Additional factors influencing immune reconstitution include baseline body mass index (BMI), with a higher BMI prior to ART initiation being associated with better CD4+ T cell recovery, and genetic polymorphisms; however, some candidate genes such as IFNL4 have not shown significant associations with immune recovery outcomes (Zhu et al., 2022; Meissner et al., 2021). Furthermore, the presence of co-infections such as tuberculosis or leishmaniasis can modulate immune responses and cytokine profiles, often leading to further CD4+ T-cell depletion and immune dysregulation (Karnan et al., 2024; Abdullahi et al., 2020).

At the molecular level, the roles of autophagy and programmed cell death pathways (apoptosis, necroptosis, ferroptosis, pyroptosis) in CD4+ T-cell homeostasis have gained attention. Dysregulation of these processes in HIV infection contributes to insufficient production and excessive destruction of CD4+ T cells, thereby impairing immune reconstitution despite ART (de Oliveira Duarte et al., 2025). Additionally, soluble immunoregulatory proteins, including immune checkpoint molecules such as ICOSL and OX40, are elevated in patients with poor CD4+ T-cell recovery and may serve as biomarkers or therapeutic targets to improve immune restoration (Moar et al., 2024).

The complex interplay between HIV and CD4+ T-cell subsets extends to the modulation of regulatory T cells (Tregs) and T helper 17 (Th17) cells, which are critical for maintaining immune homeostasis. HIV infection can increase the immunosuppressive activity of Tregs without significantly reducing their numbers, contributing to immune dysfunction. The imbalance between Treg and Th17 cells further exacerbates immune dysregulation and may influence disease progression and response to therapy (Tolomeo and Cascio, 2024; Leng et al., 2023).

### 2.2 Chronic immune activation and inflammatory microenvironment

HIV infection is characterized by persistent activation of both the innate and adaptive immune systems, leading to a chronic inflammatory state that profoundly influences the tumor microenvironment (TME). This chronic immune activation is marked by elevated levels of pro-inflammatory cytokines and chemokines in the plasma, including interleukin-6 (IL-6), tumor necrosis factor-alpha (TNF-α), and interferon-alpha (IFN-α). These soluble mediators are not only biomarkers of immune dysregulation but also active participants in shaping the inflammatory milieu that supports tumorigenesis in people living with HIV (PWH) (Dandachi and Morón, 2020; Chiu et al., 2024). HIV-1 Vpr has been implicated in inducing IL-6 production, correlating with tumor progression and malignancy risk (Chiu et al., 2024).

Chronic immune activation promotes the recruitment and infiltration of immune cells, particularly macrophages and dendritic cells, into the TME. Tumor-associated macrophages (TAMs), often characterized by high expression of the mannose receptor (CD206), exhibit functional plasticity and can be reprogrammed from pro-inflammatory to immunosuppressive phenotypes that facilitate tumor progression. These TAMs release reactive oxygen species (ROS) and reactive nitrogen species (RNS), which induce DNA damage and genomic instability in surrounding cells, thereby promoting oncogenic transformation and tumor evolution (Fiani et al., 2020). The oxidative stress induced by HIV-1 proteins, such as reverse transcriptase (RT), further exacerbates mitochondrial dysfunction and metabolic reprogramming in tumor cells, enhancing their tumorigenic and metastatic potential (Zakirova et al., 2022). This oxidative environment not only damages DNA but also modulates signaling pathways that favor tumor cell survival and proliferation.

The inflammatory microenvironment in HIV-associated malignancies is characterized by the activation of key transcription factors and signaling cascades, notably nuclear factor kappa-light-chain-enhancer of activated B cells (NF-κB) and signal transducer and activator of transcription 3 (STAT3). Activation of the NF-κB and STAT3 pathways by pro-inflammatory cytokines leads to enhanced tumor cell proliferation, angiogenesis, and metastatic dissemination, establishing a positive feedback loop that perpetuates inflammation and tumor progression (Dandachi and Morón, 2020). HIV-1 proteins such as Tat and p17 have been shown to dysregulate immune cell functions and promote aberrant angiogenesis through chemokine-like activities, further contributing to a highly angiogenic tumor microenvironment (TME) with increased microvessel density compared to sporadic tumors (Dandachi and Morón, 2020; Isaguliants et al., 2021). This angiogenic switch is critical for tumor growth and metastasis, providing nutrients and oxygen to expanding tumor masses.

Moreover, the persistent inflammatory state driven by HIV infection impairs normal immune surveillance and fosters an immunosuppressive TME. The chronic release of cytokines and chemokines recruits immunosuppressive cells, including regulatory T cells and myeloid-derived suppressor cells, which inhibit effective anti-tumor immune responses. Extracellular vesicles (EVs) derived from tumor and immune cells in the TME carry high mannose glycans that target TAMs via CD206, modulating their phenotype and function to support tumor immune evasion (Fiani et al., 2020). This intercellular communication mediated by EVs represents a novel mechanism by which chronic inflammation and immune activation in HIV infection promote tumor progression.

### 2.3 Immune checkpoint dysregulation and T-cell exhaustion

HIV infection profoundly disrupts immune homeostasis, particularly through the dysregulation of immune checkpoint molecules and the induction of T-cell exhaustion, which are pivotal in the pathogenesis of HIV-associated malignancies. Immune checkpoints such as programmed cell death protein 1 (PD-1), cytotoxic T-lymphocyte-associated protein 4 (CTLA-4), T-cell immunoglobulin and mucin domain-containing protein 3 (TIM-3), and lymphocyte activation gene 3 (LAG-3) are normally critical regulators of immune tolerance and activation. In the context of chronic HIV infection, these molecules become persistently overexpressed on both CD8+ and CD4+ T cells, driving a state of functional exhaustion characterized by diminished proliferative capacity, reduced cytokine production, and impaired cytotoxic activity. This phenomenon accelerates the decline of effective T-cell-mediated immune surveillance against tumors and opportunistic infections, thereby facilitating oncogenesis and tumor progression in PLWH.

The sustained high expression of PD-1 on T cells during HIV infection is particularly notable. PD-1 engagement with its ligand PD-L1, which is often upregulated in the tumor microenvironment, leads to inhibitory signaling that suppresses T-cell effector functions. This axis is critically involved in the immune escape mechanisms of HIV-associated lymphomas, especially non-Hodgkin lymphomas (NHL) and other AIDS-defining cancers. Studies have demonstrated that the activation of the PD-1/PD-L1 pathway correlates with poor prognosis and an aggressive disease course in HIV-related lymphomas, underscoring its role in tumor immune evasion. Moreover, other checkpoint molecules such as CTLA-4, TIM-3, and LAG-3 contribute synergistically to the exhaustion phenotype, compounding the immune dysfunction.

T-cell exhaustion in HIV infection is not merely a marker of immune dysfunction but also a driver of impaired anti-tumor immunity. Exhausted T cells lose their ability to effectively recognize and kill tumor cells and exhibit a diminished response to novel tumor antigens, compromising the immune system's capacity to control oncogenic viruses such as EBV and KSHV. These viruses are implicated in the majority of HIV-associated lymphomas, and their unchecked proliferation in the context of T-cell exhaustion promotes lymphomagenesis. The chronic antigenic stimulation by HIV and co-infecting oncogenic viruses perpetuates the expression of immune checkpoints, creating a vicious cycle of immune suppression and tumor progression.

Therapeutically, immune checkpoint inhibitors (ICIs) targeting the PD-1/PD-L1 axis have shown promise in HIV-associated lymphomas. Clinical data indicate that agents such as pembrolizumab can elicit meaningful responses in relapsed or refractory HIV-associated non-Hodgkin lymphomas, including primary effusion lymphoma and diffuse large B-cell lymphoma subtypes. These treatments can partially restore T-cell function and enhance anti-tumor immunity without exacerbating HIV replication or causing severe immune-related adverse events in most cases. The combination of ICIs with other immunomodulatory agents, such as pomalidomide, further improves outcomes by modulating the tumor microenvironment and enhancing immune effector functions.

At the molecular level, HIV infection may also contribute to oncogenesis through insertional mutagenesis, as evidenced by proviral integration into genes like STAT3, which is associated with persistently infected CD4+ T cells exhibiting anti-apoptotic and cytotoxic phenotypes resembling those of HIV-associated T-cell lymphomas. This integration can influence immune checkpoint expression and T-cell exhaustion, linking viral persistence to lymphomagenesis. Furthermore, the chronic inflammatory milieu and immune dysregulation induced by HIV infection promote genetic instability and immune escape mechanisms, reinforcing the exhausted T-cell phenotype.

**Figure 2:**
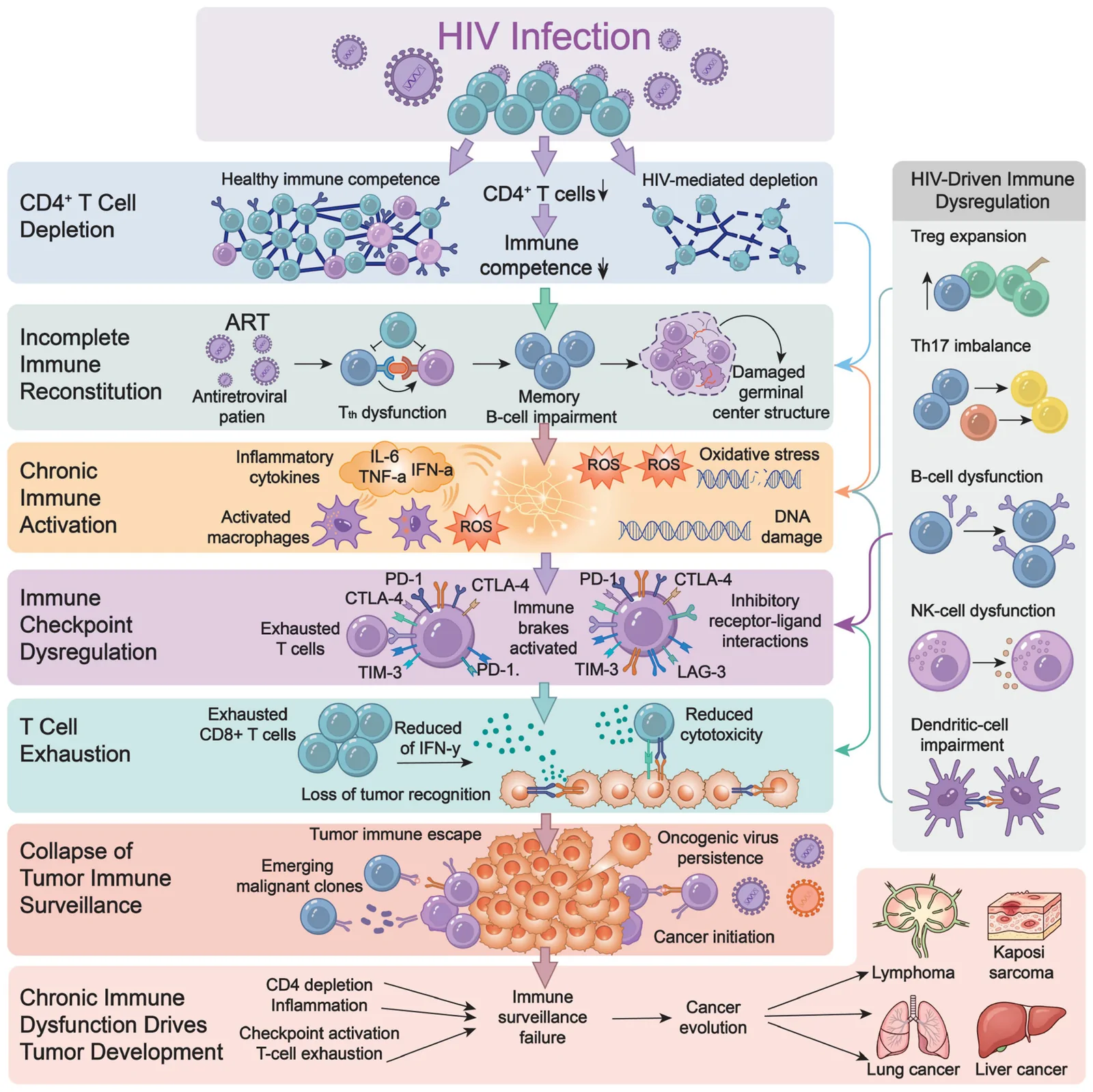
HIV-induced chronic immune dysfunction and collapse of tumor immune surveillance. HIV infection causes progressive depletion and dysfunction of CD4+ T cells, resulting in incomplete immune reconstitution despite antiretroviral therapy. Persistent immune activation promotes the production of pro-inflammatory cytokines, including IL-6, TNF-α, and IFN-α, leading to chronic inflammation and oxidative stress. Sustained antigenic stimulation induces the upregulation of inhibitory immune checkpoints, including PD-1, CTLA-4, TIM-3, and LAG-3, driving T-cell exhaustion and loss of effective antitumor immunity. These processes collectively compromise immune surveillance and create a permissive environment for malignant transformation.

In summary, immune checkpoint dysregulation and T-cell exhaustion are central to the impaired immune surveillance observed in HIV infection, facilitating the development and progression of HIV-associated malignancies. The persistent overexpression of checkpoint molecules on T cells leads to functional impairment, reducing the capacity to control oncogenic viruses and tumor cells. This immunological dysfunction is particularly pronounced in HIV-associated lymphomas, where the PD-1/PD-L1 axis plays a critical role in tumor immune evasion and correlates with adverse clinical outcomes. Emerging immunotherapeutic strategies targeting these pathways offer promising avenues to restore immune competence and improve prognosis in this vulnerable population, highlighting the importance of integrating immune checkpoint modulation into the management of HIV-driven cancers (Lurain et al., 2022, 2021; Rist et al., 2025; Suterio et al., 2024). These interconnected processes ultimately culminate in the collapse of effective tumor immune surveillance, creating a permissive environment for malignant transformation and progression (Figure 2).

## 3 HIV viral proteins and direct oncogenic mechanisms

Beyond indirect immunosuppressive effects, HIV directly contributes to oncogenesis through multiple viral proteins, particularly Tat, Nef, and Vpr, which collectively promote inflammation, immune escape, and genomic instability.

### 3.1 Tat protein and pro-tumorigenic signaling

Beyond its extracellular signaling roles, Tat disrupts intrinsic tumor suppressor mechanisms by inhibiting the functions of critical tumor suppressor proteins such as p53 and retinoblastoma protein (Rb). This inhibition leads to dysregulation of the cell cycle, allowing uncontrolled cellular proliferation and increasing genomic instability, which are hallmarks of cancer evolution. Tat's interference with p53 and Rb pathways compromises DNA damage responses and apoptosis, facilitating the survival and expansion of genetically aberrant cells. Moreover, Tat's ability to induce oxidative stress and mitochondrial dysfunction further exacerbates genomic instability and promotes oncogenic transformation (Thangaraj et al., 2021).

Tat also modulates the immune microenvironment, impairing immune surveillance and facilitating immune evasion by tumor cells. For example, Tat downregulates major histocompatibility complex (MHC) class II molecules on B cells, reducing antigen presentation and weakening CD4+ T-cell-mediated immune responses. This immune modulation contributes to the increased incidence of B-cell lymphomas in HIV-infected individuals, despite the absence of direct HIV infection in B cells. Tat-induced alterations in gene expression in B cells include downregulation of HLA-DRB1 and HLA-DRB5, mediated through suppression of the NF-κB pathway, which may promote lymphoma immune escape and tumor progression.

In the cardiovascular context, Tat promotes pathological remodeling by inducing myocardial fibrosis through the TGF-β1-CTGF signaling cascade. Tat enhances the proliferation and viability of cardiac fibroblasts and upregulates profibrotic mediators, contributing to cardiac dysfunction observed in HIV-infected patients (Jiang et al., 2021). Similarly, Tat's impact on endothelial cells includes the induction of mitochondrial dysfunction, oxidative stress, and disruption of endothelial nitric oxide synthase (eNOS) activity, leading to vascular endothelial dysfunction, a precursor to tumor angiogenesis and metastasis.

Tat's neurotoxic properties also intersect with its pro-tumorigenic roles. By activating inflammasomes such as NLRP3 in microglia and inducing chronic neuroinflammation, Tat creates a microenvironment that may facilitate oncogenic processes in the central nervous system. The protein's ability to induce mitochondrial DNA stress and ROS accumulation further contributes to cellular damage and tumorigenic transformation (Yang X. et al., 2025).

At the molecular level, Tat's interaction with the TAR RNA element is critical for its transactivation function, and subtype-specific polymorphisms in Tat influence its binding affinity and functional potency. Variants with higher affinity for TAR may exhibit enhanced transactivation and pathogenicity, potentially affecting tumorigenic outcomes in different HIV subtypes. Additionally, Tat's stability is regulated by host factors such as the E3 ubiquitin ligase ZNF598, which protects Tat from proteasomal degradation, thereby sustaining its pathological effects, including tumor promotion (Shi et al., 2026).

Importantly, Tat persists in circulation even in patients undergoing effective antiretroviral therapy with suppressed viral loads, maintaining its capacity to modulate cellular functions and promote tumorigenesis. Circulating Tat penetrates non-infected cells, perpetuating inflammation, immune dysregulation, and oncogenic signaling pathways (Shmakova et al., 2024). This persistence underscores the need for therapeutic strategies targeting Tat to mitigate its pro-tumorigenic effects.

### 3.2 Immune evasion mechanisms of the Nef protein

The HIV-1 Nef protein is a multifunctional accessory protein that plays a pivotal role in immune evasion, viral pathogenesis, and disease progression. One of the primary mechanisms by which Nef facilitates immune escape is through the downregulation of major histocompatibility complex class I (MHC-I) molecules on the surface of infected cells. By reducing MHC-I expression, Nef effectively diminishes the recognition and killing of infected cells by cytotoxic CD8+ T lymphocytes, which are critical for antiviral immunity. This downregulation is achieved by Nef-mediated rerouting of MHC-I molecules from the plasma membrane to intracellular compartments, thereby preventing their presentation of viral and tumor-associated antigens to CD8+ T cells. This mechanism not only allows HIV-infected cells to evade immune surveillance but also contributes to the persistence of infected reservoirs despite ART (Yaseen et al., 2026). Importantly, Nef remains active within tissue reservoirs even under suppressive ART, sustaining chronic immune dysfunction and facilitating viral dissemination.

Beyond immune evasion, Nef also manipulates intracellular signaling pathways to promote infected cell survival and proliferation. Specifically, Nef activates the phosphoinositide 3-kinase (PI3K)/Akt and mitogen-activated protein kinase (MAPK) pathways, which are well-known regulators of cell growth and apoptosis inhibition. Activation of these pathways by Nef enhances cell survival signals and suppresses apoptotic pathways, thereby creating a cellular environment conducive to viral replication and persistence. This pro-survival signaling not only benefits HIV replication but may also contribute to oncogenic processes by promoting the expansion of infected or transformed cells (Isaguliants et al., 2021). The ability of Nef to modulate these pathways underscores its role as a viral factor that directly influences host cell fate decisions, linking chronic HIV infection to increased cancer risk.

In the context of HIV-associated lymphomas, Nef exerts oncogenic effects by modulating B-cell receptor (BCR) signaling and the nuclear factor kappa-light-chain-enhancer of activated B cells (NF-κB) pathway. Nef's interference with BCR signaling can dysregulate normal B-cell activation and proliferation, while its activation of NF-κB promotes transcription of genes involved in cell survival and inflammation. These combined effects facilitate the malignant transformation and clonal expansion of B cells, contributing to the pathogenesis of HIV-related non-Hodgkin lymphomas (NHL) (Isaguliants et al., 2021). This oncogenic potential of Nef highlights its dual role in both immune evasion and tumor evolution within the HIV-driven tumor ecosystem.

Furthermore, Nef's interaction with host cellular machinery extends to the modulation of autophagy, an innate antiviral defense mechanism. HIV-1 Nef counteracts autophagy by enhancing the association between Beclin 1 (BECN1), a key autophagy initiator, and its inhibitor BCL2, in a manner dependent on the E3 ubiquitin ligase Parkin (PRKN). This interaction blocks autophagy initiation and maturation, thereby preventing the degradation of viral components and promoting viral persistence (Castro-Gonzalez et al., 2021). The suppression of autophagy by Nef not only aids viral survival but may also contribute to immune dysfunction and oncogenesis by impairing cellular homeostasis.

Nef also antagonizes host restriction factors such as SERINC5, a potent inhibitor of HIV infectivity. Nef recruits the cyclin K (CycK)/cyclin-dependent kinase 13 (CDK13) complex to phosphorylate SERINC5 at serine 360, facilitating its internalization and degradation via the endocytic pathway. This antagonism enhances viral infectivity by preventing SERINC5-mediated restriction of viral particle infectivity (Chai et al., 2021). Additionally, Nef can downregulate other immune regulators such as CD4 and BST-2/tetherin, further promoting viral replication and release (Giese et al., 2020).

The evolutionary plasticity of Nef's interactions with host proteins, including its SH3 domain binding and modulation of kinases like Hck, reflects its adaptation to optimize immune evasion and viral fitness across different primate lentiviruses. Structural studies reveal that Nef employs an “R-clamp” mechanism to stabilize interactions critical for kinase activation and immune modulation, with variations observed among HIV-1 groups and simian immunodeficiency viruses (SIV) (Zhao et al., 2021). This evolutionary adaptability underscores the complexity of Nef's role in HIV pathogenesis.

Moreover, Nef's presence in EVs contributes to systemic immune dysregulation and inflammation. EVs containing Nef can induce endothelial dysfunction and propagate inflammatory signaling, linking HIV infection to cardiovascular complications and potentially to tumor-promoting microenvironments (Clauss et al., 2021). The ability of Nef to modulate both infected and bystander cells via EVs expands its impact beyond direct viral infection.

### 3.3 Vpr protein and genomic instability

The HIV-1 Vpr is a multifunctional accessory protein that plays a critical role in modulating host cellular processes, particularly those related to genomic stability and cell cycle regulation. One of the hallmark activities of Vpr is its ability to induce cell cycle arrest at the G2/M phase. This arrest is mediated through the activation of the DNA damage response (DDR) pathways, which are normally engaged to maintain genomic integrity under genotoxic stress. Vpr triggers DNA damage signaling by inducing double-strand breaks and stalling DNA replication, thereby activating key DDR markers. However, despite this activation, Vpr simultaneously represses the repair of double-strand breaks by inhibiting homologous recombination (HR) and non-homologous end joining (NHEJ), two major DNA repair pathways. This dual action—inducing DNA damage while impairing its repair—leads to the accumulation of unrepaired DNA lesions, which increases mutation frequency and promotes genomic instability over time (Li et al., 2020).

Mechanistically, Vpr hijacks the host ubiquitin-proteasome system by recruiting the DCAF1-DDB1-CUL4 E3 ubiquitin ligase complex, targeting multiple host proteins for degradation. Among these targets are DNA repair enzymes such as uracil DNA glycosylase 2 (UNG2) and single-strand selective monofunctional uracil DNA glycosylase 1 (SMUG1), which are critical components of the base excision repair (BER) pathway. Vpr-mediated degradation of UNG2 leads to a loss of uracil removal activity, thereby impairing BER and contributing to the accumulation of mutations and genomic instability. This interaction has been demonstrated in B lymphocytes, where Vpr reduces immunoglobulin class switch recombination by degrading UNG2, highlighting its broader impact on host genome maintenance and immune function (Eldin et al., 2020). The inhibition of BER by Vpr not only compromises DNA repair fidelity but also facilitates viral persistence by manipulating host cell survival and proliferation.

In addition to its effects on DNA repair enzymes, Vpr interacts with the anaphase-promoting complex/cyclosome (APC/C), a key regulator of cell cycle progression. Vpr induces the degradation of APC1, an essential scaffolding subunit of APC/C, through DCAF1-dependent ubiquitination. This degradation disrupts APC/C function, contributing to the G2/M cell cycle arrest. Interestingly, this activity is conserved among primary Vpr variants from transmitted/founder viruses, underscoring its importance in viral pathogenesis. However, APC1 degradation does not directly impact Vpr's ability to induce G2 arrest or enhance HIV-1 replication in macrophages, suggesting that Vpr's modulation of APC/C may influence other aspects of HIV-1 infection and host cell biology (Barbosa et al., 2021).

The persistent expression of Vpr in HIV-infected cells, particularly in reservoir sites such as macrophages and certain tissues, has been linked to increased tumor mutational burden and accelerated clonal evolution in HIV-associated malignancies, including lung and liver cancers. Clinical cohort studies have identified circulating Vpr protein in the blood of HIV-positive individuals, with higher detection frequencies in patients with malignancies compared to those without. The presence of Vpr correlates with elevated levels of pro-inflammatory cytokines such as IL-6, which further contribute to a pro-tumorigenic microenvironment. Multivariate analyses suggest that Vpr acts as an independent risk factor for tumor occurrence in people living with HIV, implicating its role in oncogenesis through chronic immune activation and genomic instability (Matsunaga et al., 2024).

At the molecular level, Vpr-induced DNA damage activates the ATR kinase pathway, which enforces cell cycle arrest and triggers epigenetic remodeling that enhances HIV-1 transcription and latency reactivation. Vpr segregates into two functional pools: a multimeric nuclear pool associated with chromatin and a monomeric cytoplasmic pool bound to DCAF1. The nuclear pool mediates epigenetic changes that may also influence host gene expression and genomic stability, while the cytoplasmic pool facilitates ubiquitin ligase recruitment for targeted protein degradation. This multifaceted engagement with the host DDR and chromatin landscape underscores Vpr's central role in manipulating host cell fate and promoting viral persistence.

Furthermore, Vpr's interference with DNA repair and cell cycle regulation has downstream consequences for cellular senescence and tissue pathology. For example, recombinant Vpr administration in murine models induces retrotransposition of long interspersed element-1 (L1) in cardiac tissue, leading to cellular senescence and fibrosis. This suggests that Vpr-mediated genomic instability extends beyond immune cells to affect non-lymphoid tissues, potentially contributing to comorbidities such as cardiovascular disease in HIV-infected individuals (Ueno et al., 2020). Similarly, in kidney tubular epithelial cells, Vpr expression induces DNA damage responses, aberrant cell division, and progressive fibrosis, recapitulating features of HIV-associated nephropathy (HIVAN) and chronic kidney disease (Chen et al., 2023).

Collectively, Vpr induces genomic instability through G2/M cell cycle arrest, simultaneous activation and repression of DNA repair pathways, and targeted degradation of key repair proteins, fostering oncogenic transformation. These findings highlight Vpr as a potential therapeutic target to mitigate cancer evolution in HIV infection.

## 4 Immune cell reprogramming in the tumor microenvironment

HIV infection profoundly reshapes the tumor immune microenvironment through extensive immune cell reprogramming involving macrophages, dendritic cells, NK cells, T cells, and B cells.

### 4.1 Macrophage polarization and tumor-associated macrophages

HIV infection profoundly influences macrophage polarization, skewing these immune cells toward the M2 phenotype, which is characterized by anti-inflammatory and pro-tumorigenic functions. This polarization shift is marked by increased expression of surface markers such as CD163 and CD206, along with elevated secretion of the immunosuppressive cytokine interleukin-10 (IL-10). Concurrently, markers associated with the M1 phenotype, including inducible nitric oxide synthase (iNOS) and TNF-α, are downregulated, reflecting a diminished pro-inflammatory and anti-tumor response. This M2 polarization fosters an immunosuppressive TME that facilitates tumor progression and immune evasion. TAMs, predominantly of the M2 subtype in HIV-related tumors, contribute to tumor growth and metastasis through multiple mechanisms. They secrete vascular endothelial growth factor (VEGF), matrix metalloproteinases (MMPs), and transforming growth factor-beta (TGF-β), which collectively promote angiogenesis, extracellular matrix remodeling, and suppression of effective anti-tumor immunity. Recent advances in single-cell RNA sequencing (scRNA-seq) have revealed that macrophage populations within HIV-associated tumors are highly heterogeneous, with distinct subpopulations exhibiting unique transcriptional profiles and functional states. Notably, subsets expressing SPP1 (osteopontin) and TREM2 (triggering receptor expressed on myeloid cells 2) have been identified and are associated with poor clinical outcomes. TREM2+ macrophages, for instance, have been implicated in fostering an immunosuppressive milieu by modulating signaling pathways such as TLR4/Akt, which contribute to tumor radioresistance and immune escape. Therapeutic targeting of TREM2 has shown promise in preclinical models by repolarizing macrophages toward an M1 phenotype, enhancing cytotoxic T-cell infiltration, and reducing immunosuppressive cytokine secretion. However, the complexity of TREM2 signaling and its context-dependent effects necessitate further investigation to optimize therapeutic strategies. Understanding the dynamic interplay between HIV infection, macrophage polarization, and tumor evolution is critical for developing novel immunotherapeutic approaches that can reprogram the TME from a pro-tumor to an anti-tumor state, thereby improving clinical outcomes for HIV-infected cancer patients (Zhu et al., 2025; Qiu et al., 2024; Jung et al., 2022; Pavlov et al., 2026; El-Guindy et al., 2022; Giordano et al., 2024).

### 4.2 Dendritic cell dysfunction

HIV infection profoundly impairs dendritic cell (DC) populations, both quantitatively and functionally, critically undermining the host's immune surveillance and response capabilities. A hallmark of HIV pathogenesis is the reduction in the number of circulating and tissue-resident DCs, including both conventional dendritic cells (cDCs) and plasmacytoid dendritic cells (pDCs), accompanied by significant functional defects. These defects include diminished antigen uptake, processing, and presentation abilities, as well as decreased expression of essential co-stimulatory molecules such as CD80 and CD86, which are pivotal for effective T-cell priming and activation. The impairment of these functions compromises the initiation of adaptive immune responses, facilitating viral persistence and progression to AIDS. Specifically, HIV-infected individuals exhibit a marked decrease in DC capacity to capture and present antigens, compounded by the downregulation of co-stimulatory molecules necessary for T-cell activation, leading to suboptimal T-cell responses and immune exhaustion (Li et al., 2025). This functional impairment is not limited to cDCs; pDCs, crucial producers of type I interferons (IFN-I), also display altered behavior during HIV infection. Persistent activation of pDCs results in sustained IFN-α production, contributing to chronic immune activation and T-cell exhaustion, phenomena that exacerbate immune dysfunction and impede viral clearance (Li et al., 2025). This chronic immune activation state is a key driver of HIV pathogenesis and is associated with increased morbidity even in the era of cART (Mohamed et al., 2025). In the tumor microenvironment, often characterized by immunosuppressive conditions, DC dysfunction is further exacerbated. The impaired antigen-presenting capacity of DCs within tumors weakens the induction of antitumor T-cell responses, facilitating immune evasion by malignant cells. This is particularly relevant in HIV-associated malignancies, where the combined effects of HIV-induced DC dysfunction and tumor-mediated immunosuppression synergize to promote cancer progression. For instance, blastic plasmacytoid dendritic cell neoplasm (BPDCN), a malignancy derived from pDCs, exemplifies how aberrant DC function and differentiation contribute to oncogenesis and immune escape. Moreover, the tumor microenvironment often induces tolerogenic DC phenotypes, characterized by reduced co-stimulatory molecule expression and increased production of immunosuppressive cytokines, further dampening effective T-cell-mediated antitumor immunity (Wang et al., 2020). The persistent dysfunction of DCs in HIV infection and cancer underscores the critical need for therapeutic strategies aimed at restoring DC function. Recent advances in nanoparticle-based vaccine platforms have demonstrated the potential to activate both conventional and plasmacytoid DCs, enhancing antigen presentation and promoting robust CD8+ T-cell responses, which are essential for effective tumor control and viral clearance (Butkovich et al., 2023). Additionally, targeting the chronic activation and exhaustion of pDCs, for example, through depletion or blockade of IFN-I signaling, has been shown to rescue stem-like memory CD8+ T-cell responses in HIV infection, suggesting a promising avenue to restore immune competence (Li et al., 2025). Collectively, these findings highlight the multifaceted roles of DC dysfunction in HIV pathogenesis and tumor immune evasion, emphasizing the importance of therapeutic interventions that can reverse DC impairment to improve immune control of both HIV and HIV-associated cancers. Recent single-cell analysis in non-HIV-associated esophageal squamous cell carcinoma further shows that TFAM-mediated dendritic-cell programs influence immunochemotherapy response (Chen et al., 2026), supporting investigation of analogous DC states in HIV-associated tumors.

### 4.3 NK cell exhaustion and dysfunction

HIV infection profoundly alters the natural killer (NK) cell compartment, leading to exhaustion and functional impairment that contribute to immune dysregulation and tumor progression in HIV-associated malignancies such as Kaposi sarcoma (KS). One hallmark of NK cell perturbation in HIV is the shift in NK cell subset distribution, characterized by a reduction in the CD56^∧^dim cytotoxic subset and a relative increase in the CD56^∧^bright regulatory subset. The CD56^∧^dim NK cells are primarily responsible for direct cytotoxicity against infected or transformed cells, while CD56^∧^bright NK cells mainly produce immunoregulatory cytokines. This shift toward the CD56^∧^bright phenotype diminishes the overall cytolytic capacity of the NK cell pool, impairing the innate immune response to HIV-infected cells and tumor cells (Chretien et al., 2021).

At the molecular level, HIV infection modulates the expression of activating and inhibitory receptors on NK cells, further compromising their function. Activating receptors such as NKG2D and NKp30, which recognize stress-induced ligands on target cells, are downregulated in HIV-infected individuals. Conversely, inhibitory receptors including NKG2A and killer immunoglobulin-like receptors (KIRs) are upregulated, tipping the balance toward NK cell inhibition. This receptor expression pattern leads to diminished NK cell-mediated cytotoxicity and impaired antibody-dependent cellular cytotoxicity (ADCC), both critical mechanisms for controlling HIV and associated tumors (Chretien et al., 2021). Notably, the inhibitory receptor TIGIT is elevated on NK cells during untreated HIV infection, marking a mature and adaptive NK cell subset with altered functional responses; however, blocking TIGIT does not restore NK cell activity against HIV-infected targets, indicating complex regulatory pathways underlying NK cell exhaustion (Vendrame et al., 2020).

The functional consequences of NK cell exhaustion are particularly evident in HIV-associated KS, where NK cell dysfunction correlates with tumor progression and metastasis. Studies in acute myeloid leukemia (AML) patients, a malignancy with parallels to KS in terms of NK cell impairment, have identified an accumulation of unconventional CD56^−*CD*16^+ NK cells with reduced expression of activating receptors such as NKp30, NKp46, NKG2D, DNAM-1, and CD96. These dysfunctional NK cells are associated with poor clinical outcomes, suggesting that similar NK cell phenotypes may contribute to immune evasion in HIV-related KS (Clayton et al., 2021). Furthermore, HIV-infected macrophages, which serve as viral reservoirs, resist NK cell-mediated killing by skewing NK responses toward cytokine production rather than cytolytic activity, thereby preserving inflammatory responses while allowing persistence of infected cells that may promote tumorigenesis (Clayton et al., 2021).

The interplay between NK cell exhaustion and HIV-driven oncogenesis is also influenced by soluble factors and immune activation markers. Elevated levels of systemic immune activation markers such as sCD163, sCD25/IL-2Rα, and sCD40/TNFRSF5 correlate with increased angiogenic growth factors like VEGF and FGF acidic in HIV- and HHV-8-co-infected individuals, implicating chronic immune activation and NK cell dysfunction in KS pathogenesis (Nana et al., 2023). Additionally, HIV viral proteins including gp120, Nef, p17, Tat, and reverse transcriptase exert direct oncogenic effects and induce oxidative stress, further impairing NK cell function and facilitating tumor progression even in the context of ART (Isaguliants et al., 2021).

Despite ART-mediated viral suppression, NK cell exhaustion persists. Approaches such as harnessing adaptive NK cell subsets, modulating receptor expression, and targeting immune checkpoints hold promise for restoring NK cell-mediated clearance (Alrubayyi et al., 2020). Moreover, understanding the mechanisms of NK cell dysfunction in HIV-associated KS may inform the development of immunotherapies aimed at reversing NK cell exhaustion and improving clinical outcomes in this vulnerable population.

## 5 Chronic antigen stimulation and malignant transformation of B cells

### 5.1 B-cell hyperactivation and abnormal germinal center responses

HIV infection profoundly disrupts B-cell homeostasis, leading to persistent B-cell hyperactivation characterized by polyclonal proliferation, hypergammaglobulinemia, and autoantibody production. This chronic activation is a hallmark of HIV pathogenesis and contributes to immune dysfunction and lymphomagenesis. Clinically, PLWH exhibit elevated serum immunoglobulin levels and expanded B-cell subsets, including activated and exhausted phenotypes, reflecting ongoing immune stimulation despite ART (de Carvalho et al., 2021). The underlying mechanisms involve both direct and indirect effects of HIV on B cells, including viral proteins such as Tat and Nef that modulate B-cell function and survival, as well as microbial translocation-induced chronic inflammation that sustains B-cell activation (Epeldegui and Hussain, 2020).

A critical site of B-cell maturation and selection is the germinal center (GC), where B cells undergo somatic hypermutation (SHM) and class switch recombination (CSR) under the guidance of follicular helper T cells (Tfh). In HIV infection, GC responses are dysregulated, with abnormalities in Tfh function and B-cell receptor (BCR) diversification. Notably, HIV infection leads to an increased frequency of activation-induced cytidine deaminase (AID) expression in B cells, an enzyme essential for SHM and CSR but also implicated in off-target mutagenesis and lymphomagenesis (Shponka et al., 2020). Studies comparing diffuse large B-cell lymphoma (DLBCL) tissues from HIV-positive and HIV-negative patients have demonstrated higher AID expression in HIV-associated DLBCL regardless of cell-of-origin subtype, suggesting that chronic AID activity driven by HIV contributes to malignant transformation (Shponka et al., 2020). This is supported by evidence that aberrant SHM and CSR, mediated by AID, cause recurrent mutations and chromosomal translocations in DLBCL, promoting oncogenesis.

The GC microenvironment is further altered in HIV infection by perturbations in Tfh cells. Follicular helper T cells are essential for providing help to B cells during affinity maturation. HIV infection impairs Tfh function through multiple mechanisms, including the incorporation of PD-L1 into HIV virions that suppress Tfh proliferation and IL-21 production, thereby reducing B-cell help and antibody responses (Munoz et al., 2022). Moreover, follicular regulatory innate lymphoid cells (ILCFR) have been identified in human tonsils and lymph nodes, which inhibit Tfh-mediated B-cell help via TGF-β production. These cells are increased in chronic inflammatory conditions such as HIV infection, potentially contributing to impaired GC responses (O'Connor et al., 2021). Additionally, HIV infection is associated with increased frequencies of CXCR3+ Tfh cells, which correlate with HIV-specific antibody development but also serve as preferential targets for HIV infection within follicles, further complicating GC dynamics (Mitchell et al., 2025).

B-cell subsets in HIV infection also display abnormal distributions. Immunological non-responders (INRs) to ART show increased follicular and memory B cells but decreased plasma cells in the gut mucosa, indicating impaired differentiation within GCs (Wang et al., 2024). Peripheral blood studies reveal expansions of atypical memory B cells and transitional B cells with high turnover rates during early HIV infection, which correlate with disease progression. These dysfunctional B-cell populations exhibit altered expression of BAFF receptors and are influenced by elevated B-cell activating factor (BAFF) levels, which are persistently increased in HIV infection and contribute to B-cell dysregulation and chronic inflammation.

In the context of HIV-associated DLBCL, both germinal center B-cell-like (GCB) and activated B-cell-like (ABC) subtypes exhibit high-frequency mutations characteristic of aberrant GC processes. HIV-related DLBCL often presents with distinct molecular features, including high AID expression and altered BCL2 and MYC oncogene regulation, reflecting the impact of chronic immune activation and viral co-infections such as EBV. The interplay between HIV and EBV viral proteins, such as HIV Tat and EBV Zta, can downregulate antigen presentation molecules (HLA-ABC) on B cells, facilitating immune evasion and promoting lymphomagenesis.

Therapeutically, understanding the dysregulated GC responses in HIV infection has implications for vaccine design and immunotherapy. Strategies to induce cytotoxic Tfh cells within follicles, such as CD137 agonist antibody treatment, have shown promise in reducing retroviral reservoirs in murine models, highlighting the potential to modulate GC dynamics for HIV cure research (Malyshkina et al., 2023). Additionally, vaccine approaches targeting germline B-cell receptors and promoting long-lived GC responses are being explored to elicit broadly neutralizing antibodies against HIV.

### 5.2 EBV and HIV synergistic oncogenesis

The synergistic interaction between chronic HIV infection and EBV reactivation establishes a highly permissive environment for B-cell malignant transformation and lymphoma evolution.

#### 5.2.1 HIV infection leads to EBV reactivation and increased viral load; EBV latent membrane protein 1 (LMP1) and Epstein-Barr nuclear antigen 2 (EBNA2) promote B-cell proliferation and transformation via NF-κB and JAK/STAT pathways

HIV infection profoundly disrupts immune homeostasis, leading to chronic immunosuppression that facilitates the reactivation of latent viruses, including EBV. EBV, a ubiquitous gamma-herpesvirus, establishes lifelong latency primarily in B lymphocytes. In the context of HIV-induced immune dysfunction, EBV reactivation is characterized by increased viral replication and elevated viral loads, contributing to oncogenic processes. The molecular mechanisms underlying EBV-driven oncogenesis in HIV-infected individuals involve key viral latent proteins, notably latent membrane protein 1 (LMP1) and Epstein-Barr nuclear antigen 2 (EBNA2). LMP1 functions as a constitutively active mimic of the tumor necrosis factor receptor family member CD40, leading to persistent activation of the NF-κB signaling pathway. This activation promotes B-cell proliferation, survival, and resistance to apoptosis, thereby facilitating malignant transformation. Concurrently, EBNA2 acts as a transcriptional activator that modulates both viral and host gene expression, including the upregulation of oncogenes and immune evasion molecules. EBNA2 also activates the JAK/STAT pathway, further enhancing B-cell proliferation and survival. The synergistic activation of the NF-κB and JAK/STAT pathways by LMP1 and EBNA2 creates a microenvironment conducive to B-cell transformation and tumorigenesis. In HIV-infected patients, impaired immune surveillance fails to control EBV-infected B cells, allowing these oncogenic processes to proceed unchecked. This interplay between HIV-mediated immunosuppression and EBV latent protein activity underscores the molecular basis for increased EBV-associated malignancies in this population (Epeldegui and Hussain, 2020; Shindiapina et al., 2020; Furlan et al., 2020).

#### 5.2.2 EBV infection cooperates with MYC gene translocation to drive tumorigenesis in HIV-associated burkitt lymphoma

Burkitt lymphoma (BL) is a highly aggressive B-cell malignancy frequently associated with EBV infection, particularly in the context of HIV-induced immunodeficiency. The pathogenesis of HIV-associated BL involves a complex interplay between viral oncogenesis and host genetic alterations. A hallmark genetic event in BL is the translocation of the MYC oncogene, typically t(8;14)(q24; q32), which places MYC under the control of immunoglobulin heavy chain enhancers, resulting in its constitutive overexpression. EBV infection synergizes with MYC translocation to drive lymphomagenesis through multiple mechanisms. EBV latent proteins, including LMP1 and EBNA2, promote B-cell proliferation and survival, providing a proliferative advantage to cells harboring MYC translocations. Moreover, EBV infection modulates the tumor microenvironment by inducing cytokine dysregulation and immune evasion, further supporting malignant B-cell expansion. Studies have demonstrated that the majority of HIV-associated BL cases exhibit EBV positivity alongside MYC gene rearrangements, highlighting the cooperative oncogenic role of EBV and MYC in this lymphoma subtype. The presence of EBV may also influence the immunophenotypic and clinical features of BL, contributing to its aggressive behavior in HIV-infected individuals. This cooperation between viral infection and genetic aberration exemplifies the multifactorial nature of HIV-related lymphomagenesis and underscores the importance of targeting both viral and host factors in therapeutic strategies (Epeldegui and Hussain, 2020; Pather et al., 2021; Rieken et al., 2021).

#### 5.2.3 Persistent viral antigen stimulation in EBV-positive tumors leads to T-cell exhaustion and immune escape

EBV-positive tumors in HIV-infected patients are characterized by the continuous expression of viral antigens that chronically stimulate the host immune system, particularly T lymphocytes. This persistent antigenic stimulation results in T-cell exhaustion, a state of functional impairment marked by reduced proliferative capacity, diminished cytokine production, and upregulation of inhibitory receptors such as PD-1, CTLA-4, and TIM-3. The exhausted T-cell phenotype compromises effective immune surveillance and cytotoxic responses against EBV-infected malignant cells, facilitating immune escape and tumor progression. In the context of HIV infection, where immune dysregulation is already profound, the exhaustion of EBV-specific T cells is exacerbated, further impairing the host's ability to control EBV-driven oncogenesis. Additionally, EBV employs multiple immune evasion strategies, including downregulation of major histocompatibility complex molecules and secretion of immunomodulatory viral proteins, which synergize with T-cell exhaustion to promote tumor immune escape. The net effect is a tumor microenvironment that favors malignant cell survival and proliferation despite the presence of an apparently active immune system. Therapeutic interventions targeting immune checkpoints and restoring T-cell function hold promise in reversing exhaustion and enhancing anti-tumor immunity in EBV-positive malignancies associated with HIV (Epeldegui and Hussain, 2020; Shindiapina et al., 2020; Rieken et al., 2021).

### 5.3 Lymphoma clonal evolution and heterogeneity

HIV-associated lymphomas exhibit pronounced clonal heterogeneity, characterized by diverse subclonal populations, distinct mutational landscapes, and epigenetic modifications that collectively contribute to tumor complexity and evolution. This heterogeneity reflects the dynamic interplay between chronic immune dysfunction induced by HIV infection and the oncogenic processes driving lymphoma development. Studies have demonstrated that HIV-related lymphomas, particularly DLBCL and plasmablastic lymphoma (PBL), harbor a spectrum of genetic alterations, including mutations in key signaling pathways such as B-cell receptor (BCR) signaling and nuclear factor kappa-light-chain-enhancer of activated B cells (NF-κB) pathways. These mutations are not uniformly distributed but rather present in subclonal branches, indicating ongoing clonal diversification and selection pressures within the tumor microenvironment. For instance, the presence of MYD88^∧^L265P mutations, a classical lymphoma driver mutation, has been identified early in the clonal expansion of self-reactive B cells in patients with neuropathies linked to lymphoma, underscoring the role of somatic mutations in clonal evolution and disease pathogenesis (Kelly et al., 2025). Moreover, HIV integration itself can influence clonal expansion; integration sites within genes such as STAT3 have been implicated in driving preferential T-cell growth, suggesting a direct viral contribution to clonal selection and lymphoma genesis (Yoon et al., 2020), The heterogeneity extends beyond genetic mutations to include epigenetic alterations and variable expression of viral proteins like HIV-1 matrix protein p17, which is associated with increased microvascular density and may influence the tumor microenvironment and progression (Feng et al., 2022). This complex clonal architecture poses significant challenges for diagnosis and treatment, as subclonal diversity can lead to differential responses to therapy and contribute to disease relapse.

Single-cell sequencing technologies have been instrumental in elucidating the clonal evolution pathways in HIV-associated lymphomas. By profiling individual cells, these studies have mapped the trajectory from early precursor B cells to mature malignant clones, revealing critical driver events such as mutations in the BCR and NF-κB signaling pathways. These pathways are central to B-cell survival and proliferation, and their dysregulation is a hallmark of lymphoma pathogenesis. Single-cell analyses have uncovered extensive intraclonal immunoglobulin V(D)J hypermutation, indicating ongoing somatic diversification within tumor clones (Kelly et al., 2025). This diversification is accompanied by the acquisition of additional mutations in genes like CXCR4, IGLL5, and BTG2, which may modulate tumor behavior and immune evasion. The identification of such mutations at the single-cell level provides insights into the temporal sequence of mutational events and highlights potential therapeutic targets. Furthermore, single-cell studies have revealed that some driver mutations, such as MYD88^∧^L265P, are present not only in malignant clones but also in some polyclonal B cells, suggesting a complex interplay between malignant transformation and immune dysregulation (Kelly et al., 2025). These findings underscore the importance of high-resolution molecular profiling to capture the full spectrum of clonal heterogeneity and evolution in HIV-related lymphomas.

Clonal evolution in HIV-associated lymphomas is closely linked to treatment resistance and disease relapse, necessitating the development of precision-targeted therapeutic strategies. The presence of subclonal populations harboring distinct mutations can lead to differential sensitivity to chemotherapy and immunotherapy, allowing resistant clones to survive and drive disease recurrence. For example, mutations in the NF-κB pathway have been associated with aggressive disease phenotypes and poor responses to standard regimens (Kelly et al., 2025). The dynamic nature of clonal evolution also complicates prognostication, as traditional clinical indices may not fully capture the molecular diversity influencing outcomes. Recent prognostic models incorporating immune parameters such as CD4/CD8 ratios alongside clinical factors have improved risk stratification but still lack integration of molecular heterogeneity data (Chen et al., 2022). Liquid biopsy approaches analyzing circulating tumor DNA (ctDNA) offer a promising avenue for real-time monitoring of clonal dynamics and early detection of emerging resistant clones, potentially guiding adaptive therapeutic interventions (Jamal et al., 2024). Additionally, the identification of viral and host factors driving clonal expansion, such as HIV integration sites and viral protein variants, opens avenues for targeted therapies that disrupt these oncogenic mechanisms (Messali et al., 2026). Ultimately, addressing clonal heterogeneity and evolution through comprehensive molecular profiling and personalized treatment approaches is critical to improving outcomes in HIV-associated lymphomas, reducing relapse rates, and overcoming therapeutic resistance.

Despite these advances, the application of single-cell and spatial transcriptomic approaches to HIV-associated tumors remains constrained by several limitations. Well-annotated tumor tissue from this population is scarce, particularly from settings with the highest disease burden; published cohorts are typically small (often in the single digits to low tens of patients) and cross-sectional, precluding assessment of ecosystem evolution over the course of infection, ART exposure, or treatment response. High dimensionality and batch effects across sequencing platforms complicate cross-study integration, and analytical pipelines capable of distinguishing HIV/ART-driven immune signatures from tumor-intrinsic programs are not yet standardized. Finally, translating these multi-omic discoveries into clinically actionable biomarkers or stratification tools has been slow, reflecting the broader gap between discovery-phase multi-omics and validated clinical assays.

## 6 Virus-host interactions in Kaposi's sarcoma

Kaposi sarcoma represents a prototypical example of virus-host co-evolution, where HIV-mediated immune dysfunction cooperates with HHV-8 oncogenic signaling to drive angiogenesis and tumor development.

### 6.1 HHV-8 latent infection and lytic replication

HHV-8, also known as KSHV, is a gammaherpesvirus that establishes lifelong infection characterized by a latent phase and a lytic replication phase. The interplay between HIV infection and HHV-8 reactivation is critical in the pathogenesis of Kaposi's sarcoma (KS) and other HHV-8-associated malignancies, especially in immunocompromised individuals. HIV infection promotes HHV-8 reactivation from latency to lytic replication primarily through immune suppression and the induction of inflammatory cytokines such as IL-6 and TNF-α. Elevated levels of these cytokines in HIV-positive individuals create a pro-inflammatory milieu that facilitates the transition of HHV-8 into the lytic phase, thereby increasing viral replication and dissemination. For instance, studies have demonstrated that HIV-associated exosomes containing viral RNAs like the trans-activation response element (TAR) enhance HHV-8 infectivity via activation of the epidermal growth factor receptor (EGFR) pathway in oral epithelial cells, a major site for HHV-8 transmission and replication (Chen et al., 2020). This mechanism underscores the role of HIV-induced inflammation and immune dysregulation in promoting HHV-8 lytic reactivation and spread.

During latency, HHV-8 expresses a limited set of viral proteins that maintain viral episomes and modulate host cell functions to promote cell survival and proliferation. Latent proteins such as the latency-associated nuclear antigen (LANA) and viral cyclin (vCyclin) are pivotal in this process. LANA tethers the viral genome to host chromosomes, ensuring persistence during cell division, and inhibits tumor suppressor pathways by binding and inactivating p53 and Rb, thereby promoting cellular immortalization and evasion of apoptosis (Byren et al., 2024). Similarly, vCyclin mimics cellular cyclins to dysregulate the cell cycle, further contributing to oncogenesis. The suppression of p53 and Rb by these latent proteins disrupts normal cell cycle checkpoints and DNA damage responses, facilitating the accumulation of genetic alterations and malignant transformation.

The lytic phase of HHV-8 infection is characterized by the expression of a broad array of viral genes, including those encoding the viral G protein-coupled receptor (vGPCR) and viral interleukin-6 (vIL-6), which have potent oncogenic and angiogenic properties. vGPCR constitutively activates signaling pathways such as PI3K/Akt and nuclear factor kappa-light-chain-enhancer of activated B cells (NF-κB), leading to enhanced cell survival, proliferation, and angiogenesis (Wu et al., 2023). Activation of these pathways by vGPCR promotes the secretion of VEGF and other pro-angiogenic factors, contributing to the characteristic vascular lesions of KS. Concurrently, vIL-6 acts as a viral homolog of human IL-6, stimulating autocrine and paracrine signaling that supports tumor growth and immune evasion. The combined effects of these lytic proteins create a microenvironment conducive to tumor progression by promoting neovascularization and suppressing effective immune responses.

Furthermore, the dynamic balance between latency and lytic replication is influenced by host factors and viral regulatory proteins. For example, the forkhead box O (FoxO) transcription factors, FoxO1 and FoxO3, are upregulated during latent infection and enhance antioxidant defenses, reducing ROS levels and maintaining latency (Lan et al., 2023). Inhibition of FoxO proteins increases intracellular ROS, triggering lytic reactivation and subsequent cell death, suggesting a potential therapeutic strategy to target latent HHV-8 reservoirs. Additionally, HIV proteins such as Tat can modulate HHV-8 replication by altering host cholesterol metabolism and suppressing enzymes like farnesyl diphosphate farnesyltransferase 1 (FDFT1), which affects viral replication dynamics (Liu Q. et al., 2025). These intricate interactions highlight the complexity of HHV-8 latency and reactivation in the context of HIV co-infection.

Immunological factors also play a crucial role in controlling HHV-8 infection and reactivation. Neutralizing antibodies targeting lytic glycoproteins such as gpK8.1 and gHgL are associated with remission of KS, indicating that humoral immunity contributes to viral control (Byren et al., 2024). However, HIV-induced immunosuppression impairs both cellular and humoral immune responses, facilitating HHV-8 reactivation and tumor development. Studies have shown that ART reduces HHV-8 viremia and restores antibody production, underscoring the importance of immune reconstitution in managing HHV-8-associated diseases (Watanabe et al., 2023). Nonetheless, persistent immune dysfunction and chronic inflammation in HIV-infected individuals continue to promote HHV-8 lytic replication and KS pathogenesis.

Beyond these protein factors, KSHV-encoded microRNAs constitute a further, non-protein-coding layer of latency control and oncogenesis. During latency, KSHV expresses a cluster of pre-miRNAs processed into mature microRNAs (miR-K12-1 through miR-K12-12) encoded within the latency-associated region (Cai et al., 2005; Gottwein et al., 2011). Several of these directly contribute to oncogenesis and immune evasion: miR-K12-1 activates NF-κB/IL-6/STAT3 signaling by repressing IκBα, functioning as a viral oncogene (Chen et al., 2016); miR-K12-11 is an ortholog of cellular miR-155 and regulates a largely overlapping target network, including the Polycomb component JARID2; and miR-K12-3 promotes cell migration, invasion, and angiogenesis via GRK2/CXCR2/AKT signaling while reinforcing viral latency (Gottwein et al., 2011). miR-K12-1,−3, and−4-3p additionally converge on caspase-3 to suppress apoptosis of infected cells, while miR-K12-7 stabilizes latency by directly targeting the lytic transactivator RTA (ORF50) (Gottwein et al., 2011). Collectively, KSHV miRNAs suppress lytic reactivation, promote proliferative and angiogenic signaling, and blunt apoptotic and immune surveillance pathways, positioning them as central, non-protein-coding drivers of KSHV-associated oncogenesis alongside LANA, vCyclin, vGPCR, and vIL-6.

### 6.2 Angiogenesis and tumor microenvironment remodeling

KS, a hallmark AIDS-associated malignancy, is characterized by aberrant angiogenesis and profound remodeling of the TME. The pathogenesis of KS is intricately linked to infection with HHV-8, which infects endothelial cells and induces a pro-angiogenic and pro-inflammatory milieu. HHV-8-infected endothelial cells secrete a variety of angiogenic factors, notably VEGF, basic fibroblast growth factor (bFGF), and MMPs, which collectively promote neovascularization and extracellular matrix remodeling. VEGF acts as a potent mitogen for endothelial cells, stimulating proliferation and new vessel formation, while bFGF further enhances endothelial cell growth and differentiation. MMPs degrade extracellular matrix components, facilitating endothelial cell migration and the structural reorganization necessary for new vessel sprouting. This abnormal vascular proliferation not only supports tumor growth by supplying nutrients and oxygen but also contributes to the characteristic spindle cell morphology and lesion formation observed in KS. The dysregulated angiogenesis driven by HHV-8 thus establishes a permissive TME that fosters tumor progression and dissemination (Wu et al., 2023; Ensoli et al., 1994).

The interplay between HIV and HHV-8 further exacerbates angiogenesis and TME remodeling in KS. The HIV transactivator protein Tat synergizes with the HHV-8-encoded vGPCR to amplify vascular permeability and inflammatory cell infiltration. Tat, secreted by HIV-infected cells, possesses intrinsic angiogenic properties, including the ability to upregulate VEGF expression and enhance endothelial cell migration. Concurrently, HHV-8 vGPCR constitutively activates signaling pathways such as phosphatidylinositol 3-kinase (PI3K)/Akt and MAPK, leading to increased secretion of pro-inflammatory cytokines and angiogenic mediators. The cooperative action of Tat and vGPCR results in heightened vascular permeability, facilitating the extravasation of inflammatory cells into the TME. This influx of immune cells, while ostensibly part of an anti-tumor response, paradoxically contributes to tumor progression by secreting additional pro-angiogenic factors and remodeling enzymes, thereby perpetuating a vicious cycle of angiogenesis and inflammation (Wu et al., 2023; Ensoli et al., 1990).

Within the KS TME, TAMs and mast cells play pivotal roles in sustaining angiogenesis and promoting tumor growth. TAMs, recruited by chemokines and cytokines secreted by both tumor and stromal cells, adopt an alternatively activated (M2) phenotype characterized by the secretion of VEGF, bFGF, and MMPs. These factors not only stimulate endothelial cell proliferation and migration but also modulate the extracellular matrix to facilitate tumor invasion. Mast cells, similarly recruited and activated within the TME, release a repertoire of pro-angiogenic mediators including histamine, tryptase, and VEGF. Their degranulation enhances vascular permeability and recruits additional inflammatory cells, further amplifying the angiogenic cascade. The combined activity of TAMs and mast cells thus accelerates the remodeling of the TME, creating a highly vascularized and immunosuppressive niche that supports KS progression. This dynamic cellular interplay underscores the complexity of the KS microenvironment, where viral oncogenes, HIV proteins, and host immune cells converge to drive pathological angiogenesis and tumor evolution (Ribatti, 2013).

### 6.3 Immune evasion and therapeutic challenges

Kaposi sarcoma (KS), a vascular neoplasm predominantly driven by HHV-8 infection, is a quintessential example of immune evasion in the context of HIV/AIDS. KS tumor cells employ multiple mechanisms to escape immune surveillance, particularly from cytotoxic T lymphocytes (CTLs) and NK cells, which are critical for controlling viral infections and tumor progression. One primary immune evasion strategy involves the downregulation of MHC-I molecules on the surface of KS cells. This downregulation impairs antigen presentation to CD8+ T cells, thereby reducing the recognition and killing of infected or transformed cells. Concurrently, KS cells secrete immunosuppressive cytokines such as IL-10 and TGF-β, which create an immunosuppressive tumor microenvironment. IL-10 inhibits the function of antigen-presenting cells and dampens T-cell responses, while TGF-β promotes regulatory T-cell differentiation and suppresses effector T-cell activity. These factors collectively contribute to the impairment of both adaptive and innate immune responses, facilitating KS progression despite the host's immune efforts (Yang et al., 2023; Koruthu et al., 2024).

ART has revolutionized the management of HIV infection and has significantly reduced the incidence and severity of AIDS-related KS by partially restoring immune competence. ART leads to increased CD4+ T-cell counts and decreased HIV viral loads, which enhance immune surveillance against HHV-8-infected cells. However, despite ART, a subset of patients continues to experience KS progression or relapse, indicating that immune restoration alone is insufficient for complete tumor control in some cases. This persistence may be attributed to residual immune dysfunction, the establishment of viral latency, and the complex interplay between KS cells and the tumor microenvironment. Consequently, combination approaches integrating ART with chemotherapy or immunotherapy are often necessary. Chemotherapeutic agents such as pegylated liposomal doxorubicin have demonstrated efficacy in inducing tumor regression and improving survival, particularly in advanced or disseminated KS. Clinical studies have reported response rates of up to 70% when chemotherapy is combined with ART, underscoring the importance of multimodal treatment strategies (Yang et al., 2023; Dalu et al., 2021; Coldiron et al., 2021).

Emerging therapeutic strategies increasingly focus on targeting the underlying viral etiology and modulating immune checkpoints to overcome immune escape. Since HHV-8 replication is central to KS pathogenesis, antiviral agents such as valganciclovir and ganciclovir have been investigated for their potential to suppress HHV-8 lytic replication. More recently, newer antivirals like maribavir have shown promise in preclinical models, although clinical data remain limited. Targeting HHV-8 replication may reduce viral load and antigenic stimulation, thereby attenuating tumor progression. Additionally, immune checkpoint inhibitors, particularly programmed death-1 (PD-1) pathway blockers like pembrolizumab, have emerged as promising agents in KS management. These agents reinvigorate exhausted T cells, restoring their cytotoxic function against tumor cells. Early-phase clinical trials have demonstrated that PD-1 blockade can induce durable responses in patients with refractory KS, including those with HIV infection. However, the use of immune checkpoint inhibitors in HIV-positive patients requires careful monitoring due to the risk of immune reconstitution inflammatory syndrome (IRIS) and other immune-related adverse events (Chimbola et al., 2021; Matiza et al., 2021; McKinnon et al., 2025) (see Section 10.1 for a consolidated discussion of immune checkpoint inhibitor safety and efficacy data in HIV-associated cancer).

The complexity of KS immune evasion is further compounded by the compartmentalization of immune responses, as evidenced by studies showing distinct T-cell profiles in the lungs of patients with pulmonary KS compared to peripheral blood. Bronchoalveolar lavage (BAL) T cells exhibit reduced inflammatory capacity and polyfunctionality, which may contribute to localized immune escape and tumor persistence in pulmonary sites. This compartmentalized immune dysfunction highlights the need for therapeutic strategies that can effectively target both systemic and tissue-specific immune deficits (Matiza et al., 2021).

### 6.4 Limitations in studying KSHV-HIV interactions and co-infection models

The mechanistic dissection of KSHV-HIV interaction remains constrained by several factors. HIV does not directly infect KSHV-infected endothelial or B-cell lineages, so most proposed synergy (e.g., HIV Tat transactivating KSHV lytic genes, or the HIV-driven cytokine milieu supporting KSHV-infected cell survival) is inferred from cell-culture systems using exogenous Tat protein or supernatant transfer rather than authentic dual infection (Ensoli et al., 1990). Primary KS tissue is also heterogeneous and typically obtained only as small diagnostic biopsies, limiting paired virological and immunological profiling (Yang J. et al., 2025). Furthermore, most mechanistic studies predate widespread ART use, and it remains unclear how well these findings generalize to ART-treated, virologically suppressed patients who nonetheless remain at elevated KS risk; patient-derived data are further confounded by variable CD4+ counts, ART regimens, and KSHV lytic/latent burden at the time of sampling, complicating causal inference between HIV-associated immune dysfunction and KSHV oncogenic activity (Palich et al., 2021).

A related barrier to mechanistic and preclinical work is the absence of a robust small-animal model that recapitulates HIV-KSHV co-infection and KS-like disease. KSHV productively infects only human cells, and no immunocompetent animal model supports natural KSHV transmission and tumorigenesis. Humanized-BLT (bone marrow/liver/thymus) mice support KSHV latent and lytic infection via physiological routes, including the oral mucosa, and reconstitute a human immune compartment permissive for HIV co-infection (Wang et al., 2014), but this model does not reproduce the endothelial/spindle cell transformation characteristic of KS and remains technically demanding, low-throughput, and costly for mechanistic dissection at scale. Non-human primate models using related rhadinoviruses offer an alternative but do not fully mirror KSHV biology or human HIV pathogenesis, while *in vitro* primary effusion lymphoma cell lines and KSHV-infected endothelial cultures allow for tractable mechanistic study but cannot capture systemic immune dysfunction or tumor-stroma interactions. Humanized-mouse and organoid/co-culture approaches therefore remain priority areas for model development in this field.

## 7 Unique characteristics of non-AIDS-defining tumors

Beyond AIDS-defining cancers, HIV infection also promotes the development of multiple organ-specific non-AIDS-defining malignancies through distinct yet partially overlapping tumor ecosystem mechanisms.

### 7.1 Lung cancer: chronic inflammation and genomic instability

Lung cancer incidence is notably elevated among PLWH, with lung adenocarcinoma being the predominant histological subtype observed in this population. This increased risk is multifactorial, involving chronic inflammation, immune dysregulation, and direct effects of HIV infection on the lung microenvironment. Chronic inflammation, a hallmark of HIV infection even under antiretroviral therapy, contributes to a pro-tumorigenic milieu by promoting oxidative stress, DNA damage, and aberrant cellular signaling pathways. The persistent immune activation and cytokine release facilitate oncogenic transformation and tumor progression. Moreover, HIV-induced immunosuppression impairs immune surveillance, allowing malignant cells to evade detection and elimination. Within the TME of HIV-associated lung adenocarcinoma, there is a characteristic reduction in cytotoxic CD8+ T-cell infiltration, which is critical for anti-tumor immunity. Concurrently, there is an increase in immunosuppressive cell populations, including Tregs and myeloid-derived suppressor cells (MDSCs), which further dampen effective immune responses and foster tumor growth. This immunosuppressive TME not only facilitates tumor immune escape but also correlates with poorer clinical outcomes. Genomic analyses of lung adenocarcinomas in HIV-infected individuals reveal a higher frequency of mutations in key oncogenes and tumor suppressor genes such as KRAS, EGFR, and TP53 compared to non-HIV-infected counterparts. These mutations contribute to genomic instability, driving tumor heterogeneity and resistance to therapy. Notably, elevated tumor mutational burden (TMB) has been observed, which may reflect the cumulative DNA damage from chronic inflammation and impaired DNA repair mechanisms in the context of HIV. The presence of these mutations and increased TMB has implications for targeted therapies and immunotherapy responsiveness. For instance, EGFR mutations, which are actionable targets in lung adenocarcinoma, show distinct prevalence and mutation patterns in HIV-positive patients, necessitating routine molecular profiling to guide personalized treatment. Furthermore, the interplay between chronic inflammation, immune suppression, and genomic alterations creates a complex tumor ecosystem that influences tumor evolution and therapeutic resistance. Recent studies employing single-cell RNA sequencing and advanced bioinformatics have begun to unravel the heterogeneity of tumor and immune cell populations within the HIV-associated lung cancer microenvironment, highlighting potential biomarkers and therapeutic targets. In summary, lung adenocarcinoma in HIV-infected patients is driven by a confluence of chronic inflammatory processes and genomic instability, compounded by an immunosuppressive TME characterized by diminished CD8+ T-cell infiltration and increased Tregs and MDSCs. Understanding these mechanisms is critical for developing effective diagnostic, prognostic, and therapeutic strategies tailored to this vulnerable population (Mo et al., 2024; Hondelink et al., 2023; Kunnumpurath et al., 2020; Rocha et al., 2023; Dhakar et al., 2022).

### 7.2 Liver cancer: viral co-infection and fibrotic microenvironment

HCC in PLWH is frequently associated with co-infection by HBV or HCV, which significantly contributes to chronic liver inflammation, fibrosis, and ultimately malignant transformation of hepatocytes. Epidemiological studies in sub-Saharan Africa and other high-prevalence regions have demonstrated that individuals co-infected with HIV and HBV present with HCC at a younger age compared to those with HBV monoinfection, exhibiting a more aggressive clinical course and poorer survival outcomes (Maponga et al., 2020; Sobnach et al., 2025). The synergistic effect of HIV and hepatitis viruses accelerates liver disease progression, as HIV-induced immunosuppression impairs viral clearance and promotes persistent hepatic inflammation. Chronic hepatitis and cirrhosis, driven by ongoing viral replication and immune-mediated hepatocyte injury, create a fibrotic microenvironment conducive to hepatocarcinogenesis (Wan et al., 2022; Brantly et al., 2025). Notably, HIV infection exacerbates liver fibrosis by activating hepatic stellate cells, the primary fibrogenic cells in the liver, thereby accelerating extracellular matrix deposition and architectural distortion (Brantly et al., 2025). This fibrotic milieu not only facilitates malignant transformation but also alters the TME, influencing immune surveillance and tumor progression.

Within the HCC TME of HIV-infected patients, immune dysfunction is a hallmark feature. CD8+ CTLs, critical for antitumor immunity, exhibit functional exhaustion characterized by diminished proliferative capacity, reduced cytokine production, and upregulation of inhibitory receptors, collectively impairing their ability to eliminate malignant hepatocytes (Gao et al., 2025). Concurrently, TAMs in the liver skew toward an M2-polarized phenotype, supporting tumor growth through immunosuppressive cytokine secretion, promotion of angiogenesis, and extracellular matrix remodeling (Brantly et al., 2025). This M2 polarization is further potentiated by viral infections, including HIV, HBV, and HCV, which modulate macrophage function and contribute to an immunosuppressive TME. The depletion of intrahepatic CD4+ T cells, observed in HIV/HBV-co-infected HCC patients, further compromises adaptive immunity and correlates with worse prognosis (Gao et al., 2025). Moreover, the presence of HBV Pre-S deletion mutants in co-infected individuals has been linked to increased microvascular invasion and capsular infiltration, indicating more aggressive tumor biology in the context of HIV co-infection (Gao et al., 2025).

Despite advances in ART and direct-acting antivirals (DAAs) for HCV, the risk of HCC remains elevated in HIV-co-infected populations, underscoring the persistent impact of chronic immune activation and fibrosis (Park et al., 2026; van Santen et al., 2026). Longitudinal studies reveal that while viral suppression improves liver fibrosis indices and reduces HCC incidence, the fibrotic microenvironment established prior to treatment initiation may sustain oncogenic processes (Vinikoor et al., 2024; Lindemann and Doyle, 2024). The interplay between viral co-infections and HIV-induced immune dysregulation thus creates a unique hepatic ecosystem that favors tumor initiation and progression.

Screening and surveillance strategies for HCC in HIV/HBV or HIV/HCV-co-infected patients are challenged by the altered natural history and earlier onset of liver cancer in this population. Current guidelines emphasize the importance of regular imaging and biomarker assessment; however, the sensitivity of ultrasound-based surveillance is limited in HIV-infected individuals due to the complex liver pathology and immune alterations (Merchante et al., 2020; Riebensahm et al., 2022). Emerging diagnostic tools, including liquid biopsy assays detecting circulating tumor DNA and methylation-based inflammation scores, show promise in improving early detection of HCC among PLWH (Kim et al., 2025; Zhang et al., 2025). Furthermore, risk stratification models incorporating immune parameters such as CD4+ T-cell counts and fibrosis scores (e.g., FIB-4) have demonstrated prognostic value in predicting survival and guiding therapeutic decisions (Zhao et al., 2022).

Therapeutically, HIV-infected HCC patients benefit from standard oncologic interventions, including surgical resection, transarterial chemoembolization (TACE), and systemic therapies, with outcomes comparable to those of HIV-negative counterparts when adjusted for tumor stage and liver function (Lu et al., 2024; Kong et al., 2021; Menon et al., 2025). However, immune dysfunction within the tumor microenvironment may influence responses to emerging immunotherapies, necessitating further research into tailored treatment approaches for this population (Donne and Lujambio, 2023). The integration of ART with antiviral therapies targeting HBV and HCV remains foundational to mitigating liver disease progression and reducing HCC risk (Vinikoor et al., 2024; Lindemann and Doyle, 2024). Additionally, addressing coexisting factors such as alcohol use, metabolic syndrome, and healthcare disparities is critical to improving outcomes in HIV-associated HCC (Lui et al., 2023; Vikash et al., 2023).

### 7.3 Anal cancer: persistent HPV infection and immune surveillance failure

Anal cancer incidence is markedly elevated among individuals infected with HIV, a phenomenon closely linked to persistent infection with high-risk HPV types. Epidemiological data demonstrate that HIV-positive populations, particularly men who have sex with men (MSM), exhibit a significantly higher risk of developing anal squamous cell carcinoma (SCC) compared to the general population, with HIV infection increasing the risk by approximately 19-fold (Eng et al., 2022). This heightened susceptibility is primarily attributed to the impaired immune surveillance caused by HIV-induced immunosuppression, which facilitates the persistence of oncogenic HPV infections that would otherwise be cleared by a competent immune system.

The pathogenesis of anal cancer in HIV-infected individuals involves the failure to eradicate HPV, leading to chronic viral persistence and subsequent oncogenic transformation. HIV infection results in a depletion of CD4+ T lymphocytes, which are crucial for mounting effective antiviral immune responses. This immunodeficiency impairs the clearance of HPV, allowing the virus to maintain a persistent presence in the anal epithelium. The oncogenic potential of HPV is mediated by its viral oncoproteins E6 and E7, which disrupt critical tumor suppressor pathways by targeting p53 and Rb for degradation. The degradation of p53 and Rb leads to uncontrolled cellular proliferation and genomic instability, fostering malignant transformation of infected epithelial cells (Bushara et al., 2022).

Moreover, the tumor microenvironment in anal cancer among HIV-infected patients is characterized by immune evasion mechanisms that further compromise antitumor immunity. Notably, there is an upregulation of programmed death-ligand 1 (PD-L1) expression within the tumor milieu. PD-L1, expressed on both tumor cells and infiltrating immune cells, interacts with the programmed death-1 (PD-1) receptor on T cells, leading to T-cell exhaustion and functional impairment. This immune checkpoint pathway effectively blunts cytotoxic T-lymphocyte responses against HPV-infected and transformed cells, facilitating tumor progression (Chowdhury et al., 2026). Studies have shown that PD-L1 expression correlates with reduced T-cell infiltration and is associated with poorer prognosis in anal SCC, underscoring the role of immune checkpoint-mediated immune escape in this malignancy.

The immunosuppressive state induced by HIV also contributes to chronic inflammation within the anal mucosa, which paradoxically promotes oncogenesis. Chronic inflammation can create a microenvironment conducive to tumor development by producing pro-inflammatory cytokines and reactive oxygen species that cause DNA damage and promote cellular proliferation. Additionally, HIV-related chronic inflammation upregulates PD-1 expression on CD8+ T cells, exacerbating T-cell exhaustion and diminishing effective antitumor responses.

Clinical studies have highlighted the importance of immune status in the progression of anal intraepithelial neoplasia (AIN) to invasive anal cancer. Individuals living with HIV who have low CD4+ counts, particularly below 200 cells/μL, exhibit a significantly increased risk of progression from high-grade AIN to anal SCC (Faber et al., 2022). This finding emphasizes the critical role of immune competence in controlling HPV-mediated neoplastic transformation. Furthermore, the presence of co-factors such as prior condyloma acuminata and HPV-16 infection further elevates the risk of developing high-grade lesions and invasive cancer in this population (Pérez-González et al., 2024).

The therapeutic landscape for anal cancer in HIV-infected patients has evolved with the recognition of the tumor's immunobiology. While chemoradiotherapy remains the standard of care for localized disease, the advent of immunotherapies targeting the PD-1/PD-L1 axis offers promising avenues for treatment, especially in advanced or metastatic settings. Clinical trials investigating PD-1 inhibitors such as retifanlimab have demonstrated durable antitumor activity in patients with previously treated anal SCC, regardless of HIV or HPV status, highlighting the potential to overcome immune evasion mechanisms. These findings suggest that immune checkpoint blockade can partially restore antitumor immunity in the context of HIV-associated immunosuppression.

Despite these advances, challenges remain in the screening and early detection of anal cancer among HIV-positive individuals. Current guidelines recommend regular anal cytology and high-resolution anoscopy screening in high-risk populations, including people living with HIV, to identify and treat precursor lesions before progression to invasive cancer. However, access to screening modalities and adherence to surveillance programs vary widely, limiting their effectiveness. Moreover, the social stigma associated with anal cancer and its risk factors often impedes timely diagnosis and intervention (Eng et al., 2022).

## 8 Tumor evolutionary pathways and clonal dynamics

### 8.1 Early clonal selection and driver mutations

The early evolutionary phase of HIV-associated tumors is characterized by a complex interplay between chronic immune pressure and a pro-inflammatory microenvironment that fosters the selection of clones with proliferative advantages. In this context, chronic immune dysfunction induced by HIV infection creates a milieu where certain genetic alterations confer survival benefits to emerging neoplastic clones. For instance, translocations involving the MYC oncogene and mutations affecting the BCR signaling pathway are frequently observed in early tumor clones, reflecting their role in driving aberrant proliferation under immune stress. These genetic events are not isolated but occur within specialized anatomical niches such as germinal centers or mucosal tissues, where tumor precursor cells accumulate mutations and diversify into multiple subclones. Single-cell sequencing technologies have elucidated this intratumoral heterogeneity, revealing a mosaic of subclonal populations that evolve under selective pressures imposed by the immune system and the inflammatory environment. Among the critical driver mutations identified, alterations in TP53 and NOTCH1 stand out due to their dual role in promoting immune evasion and genomic instability. TP53 mutations, for example, impair the cellular DNA damage response and apoptosis pathways, enabling tumor cells to survive despite genotoxic stress and immune surveillance. Similarly, NOTCH1 mutations modulate signaling pathways that influence cell differentiation and immune interactions, further facilitating tumor progression. The cumulative effect of these driver mutations is the establishment of a tumor ecosystem adept at circumventing host defenses, thereby promoting malignant evolution in the context of HIV-induced chronic immune dysfunction. This early clonal selection process underscores the importance of understanding the molecular underpinnings of HIV-associated oncogenesis, as it may inform targeted therapeutic strategies aimed at intercepting tumor evolution at its nascent stages (Matsunaga et al., 2024; Lui et al., 2023; Vikash et al., 2023; Eng et al., 2022; Bushara et al., 2022; Chowdhury et al., 2026; Faber et al., 2022; Pérez-González et al., 2024; Arora et al., 2021; Silverberg et al., 2021; Hassan and Ito, 2021).

### 8.3 Immune editing and tumor immune escape

Tumor cells undergo a dynamic process known as immune editing, which enables them to evade immune surveillance and establish progressive malignancies. This process involves the gradual loss of tumor immunogenicity through multiple mechanisms, including the downregulation of MHC-I molecules, expression of immune checkpoint ligands such as PD-L1, and secretion of immunosuppressive factors that modulate the tumor microenvironment. The downregulation of MHC-I molecules impairs antigen presentation to cytotoxic CD8+ T cells, thereby reducing tumor cell recognition and elimination. Concurrently, the upregulation of immune checkpoint ligands like PD-L1 engages inhibitory receptors such as PD-1 on T cells, leading to T-cell exhaustion and functional impairment. Additionally, tumor-derived immunosuppressive cytokines and factors, including IL-10, TGF-β, and indoleamine 2,3-dioxygenase (IDO), contribute to the suppression of effective anti-tumor immune responses and promote regulatory T-cell (Treg) expansion. These immune editing mechanisms collectively facilitate tumor immune escape, allowing malignant clones to survive, proliferate, and metastasize.

In the context of HIV infection, chronic immune dysfunction accelerates the immune editing process. HIV-induced immunodeficiency compromises the host's capacity to mount effective immune responses against emerging tumor cells. The persistent immune activation and exhaustion characteristic of chronic HIV infection lead to diminished CTL function and impaired NK cell activity, both of which are critical for tumor surveillance. Moreover, HIV infection is associated with increased expression of immune checkpoint molecules such as PD-1 on T cells and PD-L1 on tumor cells, further exacerbating immune exhaustion and facilitating tumor immune evasion. For example, studies in HIV-associated HCC have demonstrated significantly higher PD-L1 expression in tumor tissues and an immune-exhausted tumor microenvironment characterized by increased regulatory CD4+/FOXP3+ T cells and exhausted CD8+/PD-1+ T cells compared to HIV-negative counterparts (Pinato et al., 2023). This immune dysregulation impairs the clearance of malignant cells and promotes tumor progression.

The expansion of immune escape tumor clones during cancer evolution is closely linked to disease progression and metastasis. Single-cell transcriptomic analyses of circulating tumor cells (CTCs) in hepatocellular carcinoma have revealed that immune evasion mechanisms, such as the overexpression of chemokines like CCL5, facilitate the recruitment of regulatory T cells and suppress effective anti-tumor immunity, thereby enhancing metastatic potential (Sun et al., 2021). Similarly, in other malignancies, tumor cells exploit immune checkpoint pathways and metabolic reprogramming to create an immunosuppressive niche that favors the clonal expansion of immune-resistant variants. For instance, the glycosylation modification of PD-L1 mediated by guanine monophosphate synthase (GMPS) stabilizes PD-L1 expression on tumor cells, impairing CD8+ T-cell cytotoxicity and promoting immune escape in HCC (Guo et al., 2025). In renal cell carcinoma, hexokinase HK3-mediated O-GlcNAcylation of EP300 enhances PD-L1 transcription and protein stability, contributing to immune evasion and tumor progression (Zhang et al., 2024). These molecular adaptations underscore the intricate interplay between tumor evolution and immune escape.

In HIV-infected individuals, chronic immune dysfunction not only accelerates immune editing but also influences the tumor microenvironment, leading to a higher incidence and more aggressive behavior of NADCs. Epidemiological data indicate that HIV-positive patients present with malignancies at a younger age and often with advanced disease stages, reflecting impaired immune surveillance (Berman et al., 2022). The compromised immune system in HIV infection facilitates the emergence and expansion of tumor clones capable of evading immune detection, which correlates with poorer clinical outcomes and increased metastatic potential. Furthermore, the upregulation of immune checkpoint molecules in HIV-associated tumors suggests that immune escape mechanisms are potentiated in this population, highlighting the potential utility of immune checkpoint inhibitors. Early clinical evidence supports the safety and efficacy of PD-1/PD-L1 blockade in HIV-positive patients with cancers such as lung cancer, where these therapies can restore immune function and enhance anti-tumor activity (Chen et al., 2026) (discussed further in Section 10.1).

## 9 Impact of antiretroviral therapy on the tumor ecosystem

### 9.1 Immune reconstitution and reduced tumor risk

ART has revolutionized the management of HIV infection by effectively suppressing viral replication, leading to immune restoration primarily characterized by the recovery of CD4+ T-cell counts and function. This immune reconstitution is pivotal in reducing the risk of AIDS-defining malignancies such as Kaposi sarcoma (KS) and NHL, which are closely linked to profound immunosuppression in untreated HIV infection. ART-mediated viral suppression allows for the gradual restoration of CD4+ T cells, which are essential for orchestrating effective immune responses against oncogenic viruses like HHV-8 in KS and EBV in NHL. Clinical data demonstrate that patients on ART with improved CD4+ counts exhibit a marked decline in the incidence of these tumors, underscoring the protective role of immune recovery (Sudharshan et al., 2020; Imeh et al., 2023; Shabestari et al., 2026).

Beyond CD4+ T-cell restoration, ART enhances the functional capacity of cytotoxic CD8+ T lymphocytes and NK cells within the tumor microenvironment, which are critical for immune surveillance and the elimination of malignant cells. Improved CD8+ T-cell responses contribute to the recognition and clearance of virus-infected or transformed cells, while NK cells provide innate immune defense against tumor development. Studies have shown that ART leads to increased CD8+ T-cell polyfunctionality and NK cell cytotoxicity, collectively strengthening antitumor immunity (Rajdev et al., 2024). This immunological improvement is reflected in clinical outcomes where ART-treated patients demonstrate better control of KS and other HIV-associated malignancies, with some achieving partial or complete remission without additional chemotherapy (Rajdev et al., 2024; Cuellar et al., 2022).

However, immune reconstitution is not uniform across all patients. A subset of individuals, termed INRs, fails to achieve adequate CD4+ T-cell recovery despite effective viral suppression. These patients remain at elevated risk for opportunistic infections and malignancies, including AIDS-defining cancers. Factors contributing to incomplete immune reconstitution include low baseline CD4+ counts, co-infections such as tuberculosis and hepatitis B virus, poor adherence to ART, and host genetic factors influencing immune recovery dynamics (Hailu and Wasihun, 2021; Kacker et al., 2023; Zaongo and Chen, 2023). The persistence of immune activation and inflammation in INRs further impairs immune restoration and may promote oncogenesis through chronic immune dysregulation (Zaongo and Chen, 2023).

Moreover, the timing of ART initiation critically influences immune reconstitution and cancer risk. Earlier ART initiation at higher CD4+ counts is associated with a lower incidence of AIDS-defining cancers and improved survival, highlighting the importance of prompt diagnosis and treatment initiation (Silverberg et al., 2021). Conversely, delayed ART initiation or poor adherence results in sustained immunosuppression and increased tumor risk. Even among patients with immune recovery, the risk of NADCs remains elevated, possibly due to persistent immune dysfunction, chronic inflammation, and oncogenic exposures (Mremi et al., 2020).

In addition to ART, adjunctive therapies and interventions targeting immune restoration are under investigation to enhance CD4+ T-cell recovery and reduce malignancy risk. For example, immunotherapies such as immune checkpoint inhibitors have shown promise in HIV-infected patients with cancer, demonstrating safety and efficacy without compromising HIV control or CD4+ T-cell counts (Rajdev et al., 2024). Complementary approaches including traditional Chinese medicine and metformin are also being explored for their potential to modulate immune function and improve reconstitution in INRs (Zaongo and Chen, 2023; Ding et al., 2024) (see Section 10.1 for a dedicated discussion of immune checkpoint inhibitors in this setting).

### 9.2 Drug interactions and therapeutic challenges

The management of cancer in PLWH presents unique therapeutic challenges, particularly due to complex drug-drug interactions (DDIs) between ART and chemotherapeutic agents. ART regimens commonly include protease inhibitors (PIs) and integrase strand transfer inhibitors (INSTIs), which are metabolized through cytochrome P450 enzymes, especially CYP3A4. Chemotherapy drugs such as cyclophosphamide and doxorubicin (an anthracycline) also undergo hepatic metabolism involving similar enzymatic pathways. The co-administration of ART and chemotherapy can therefore lead to significant pharmacokinetic interactions that alter drug plasma concentrations, impacting both efficacy and toxicity profiles. For instance, protease inhibitors are potent CYP3A4 inhibitors and can increase the plasma levels of chemotherapeutic agents metabolized by this enzyme, potentially exacerbating toxicities such as myelosuppression and cardiotoxicity. Conversely, some ART drugs may induce CYP enzymes, reducing chemotherapy drug levels and compromising antitumor efficacy (Berretta et al., 2023; Frega et al., 2020). This necessitates careful consideration and monitoring when combining these therapies.

Moreover, certain ART drugs have intrinsic toxicities that may overlap or synergize with chemotherapy-induced adverse effects. Zidovudine (AZT), a nucleoside reverse transcriptase inhibitor, is well-known for its bone marrow suppressive effects, which can exacerbate chemotherapy-related cytopenias. This overlap increases the risk of severe neutropenia, anemia, and thrombocytopenia, complicating treatment delivery and increasing infection risk (Berretta et al., 2023). The hematologic toxicity of zidovudine may necessitate dose adjustments or substitution with less myelotoxic agents during cancer treatment. Additionally, the immunosuppressive effects of both ART and chemotherapy can compound, further impairing immune surveillance and increasing susceptibility to opportunistic infections, which are already a concern in PLWH.

Given these complexities, individualized treatment strategies are essential to balance effective viral suppression and optimal cancer control. Multidisciplinary collaboration involving oncologists, infectious disease specialists, and clinical pharmacists is critical to optimize ART and chemotherapy regimens. This includes selecting ART agents with minimal CYP450 interactions when possible, adjusting chemotherapy dosing, and closely monitoring hematologic parameters and drug levels (Berretta et al., 2023; Parra-Lara et al., 2023). For example, paclitaxel and carboplatin chemotherapy have been demonstrated to be tolerable at full doses in PLWH, even when combined with CYP3A4-inhibiting ART such as ritonavir, without significant pharmacokinetic alterations or increased toxicity (Haigentz et al., 2022). Such evidence supports the feasibility of standard chemotherapy dosing with appropriate vigilance.

Furthermore, the timing of ART initiation relative to chemotherapy is an important consideration. Early initiation or continuation of ART during cancer treatment improves overall survival and reduces AIDS-related complications but may increase the risk of DDIs and overlapping toxicities. Conversely, interruption of ART may lead to viral rebound and immune deterioration. Therefore, individualized ART adjustments, including switching to regimens with fewer interactions or toxicities, are often required during chemotherapy (Berretta et al., 2023; van der Wulp et al., 2026). Therapeutic drug monitoring and pharmacovigilance are valuable tools to guide these adjustments.

In addition to pharmacokinetic interactions, pharmacodynamic interactions must be considered. For example, glucocorticoids are frequently used in oncology for their anti-inflammatory and antiemetic properties but may impair immune function and potentially reduce the efficacy of immunotherapies. In PLWH, the immunosuppressive effects of glucocorticoids may be more pronounced, necessitating their judicious use (Prasath et al., 2025). The evolving landscape of cancer immunotherapy in PLWH also raises concerns about interactions with ART and immune status, although emerging data suggest immunotherapy can be safe and effective in this population (Khalaf et al., 2022).

Overall, individualized treatment that balances ART and chemotherapy requires multidisciplinary care, vigilant monitoring for DDIs, and expanded inclusion of PLWH in clinical trials (Berretta et al., 2023; Frega et al., 2020; Parra-Lara et al., 2023).

### 9.3 Long-term effects on the tumor microenvironment

Combination ART has revolutionized the management of HIV infection, significantly reducing viral loads and improving immune function. One of the critical benefits of ART in the context of HIV-associated malignancies is its capacity to modulate the TME by lowering chronic inflammation levels. Chronic inflammation, driven by persistent immune activation and elevated pro-inflammatory cytokines, is a well-recognized promoter of tumorigenesis and tumor progression in PLWH. ART reduces systemic inflammation by suppressing HIV replication, which in turn decreases circulating pro-inflammatory cytokines such as IL-6, TNF-α, and VEGF, key mediators of angiogenesis and tumor growth. For instance, HIV protease inhibitors (HIV-PIs), a component of ART, have been shown to inhibit tumor angiogenesis by downregulating matrix metalloproteinase-9 (MMP-9) and VEGF/VEGFR2 signaling while upregulating tissue inhibitors of metalloproteinases (TIMP-3), leading to normalization of tumor vasculature and reduced hypoxia in cervical carcinoma models (Qiu et al., 2020). This normalization enhances chemotherapy delivery and efficacy, indicating that ART not only controls HIV but also exerts direct and indirect antitumor effects by remodeling the TME.

Despite these benefits, long-term ART use is associated with metabolic abnormalities, including dyslipidemia, insulin resistance, and chronic low-grade inflammation, which may paradoxically contribute to tumor progression. Metabolic dysfunctions induced by ART can alter the TME by promoting a pro-tumorigenic milieu characterized by increased oxidative stress, altered adipokine secretion, and immune dysregulation. For example, elevated levels of galectin-9, a modulator of both inflammatory and immunosuppressive responses, have been linked to chronic HIV infection and are predictive of adverse non-AIDS events, including malignancies (Premeaux et al., 2021). These metabolic and inflammatory perturbations may sustain a tumor-permissive environment despite viral suppression, underscoring the complexity of ART's long-term impact on cancer risk and progression in PLWH.

Furthermore, studies reveal that even under effective ART, the immune cell composition and function within the TME remain abnormal. Persistent HIV reservoirs in tissues such as gut-associated lymphoid tissue (GALT) maintain a spatially organized viral microenvironment (VME) characterized by immunosuppressive cellular architectures enriched for Tregs, innate lymphoid cells, and mast cells, which resemble tertiary lymphoid structures and contribute to immune evasion (Hope et al., 2026). This immunosuppressive milieu impairs effective antitumor immune responses, facilitating tumor survival and progression. Additionally, HIV integration into tumor suppressor genes such as PTEN has been observed in lung cancer tissues from PLWH, with reduced PTEN expression correlating with areas of HIV antigen presence, suggesting that HIV may directly modulate the TME to favor oncogenesis (Smith et al., 2025).

Immune cell subset alterations are also evident in HIV-associated malignancies. For example, Kaposi sarcoma (KS) tissues from PLWH show overexpression of the oncogenic Wilms' tumor 1 (WT1) antigen, which correlates with sparse T-cell infiltration and an immune-evasive TME (Morales et al., 2024). Similarly, transcriptomic profiling of KS tumors reveals distinct immune microenvironment subtypes differing by HIV status, with HIV-positive KS exhibiting immune-enriched/fibrotic and fibrotic subtypes, along with dynamic changes in immune checkpoint gene expression over time (Yang J. et al., 2025). These findings highlight that ART, while improving systemic immunity, does not fully restore normal immune surveillance within the TME, necessitating further optimization of therapeutic strategies to enhance antitumor immunity.

Beyond its effects on the local tumor microenvironment, long-term ART use also carries systemic cardiometabolic consequences relevant to the broader HIV tumor ecosystem. People with HIV have a 1.2–2-fold higher risk of myocardial infarction and other cardiovascular events compared with HIV-negative individuals, driven by a combination of ART-associated metabolic and cardiovascular adverse effects, HIV-driven monocyte activation, endothelial dysfunction, and persistent cytokine secretion superimposed on conventional risk factors such as smoking, hypertension, and dyslipidemia (Sayad, 2026). Routine cardiovascular risk assessment and ART regimen optimization (e.g., preferring metabolically favorable, integrase-inhibitor-based regimens), alongside inflammation-modulating strategies, are increasingly emphasized in clinical guidelines. This underscores that ART, while essential for viral suppression and reducing oncogenic risk, carries its own long-term inflammatory and cardiometabolic burden that forms part of the HIV-driven tumor ecosystem and should be considered alongside cancer-focused outcomes.

## 10 New therapeutic strategies and future directions

**Figure 3:**
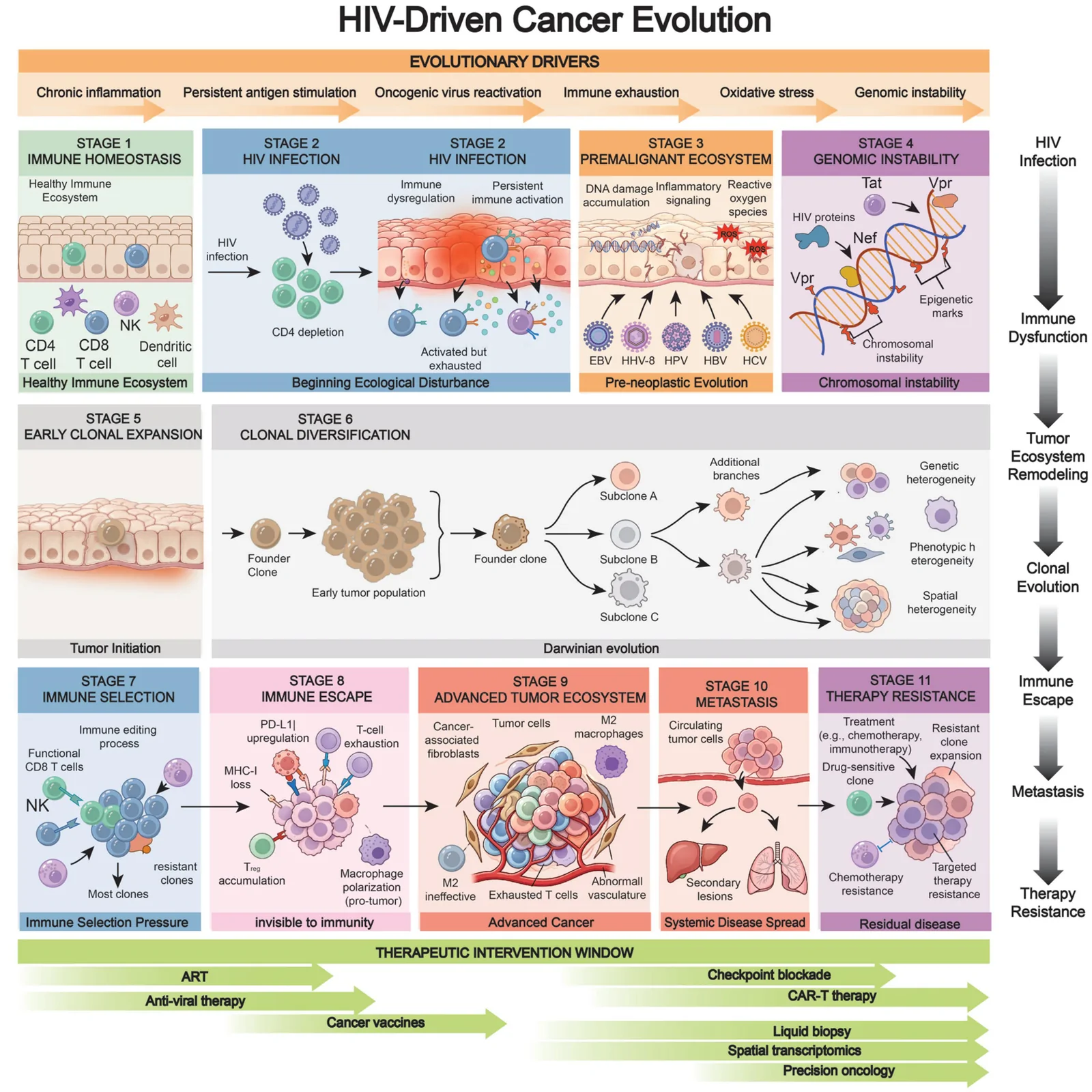
Evolutionary trajectory of HIV-driven cancer development and therapeutic resistance. HIV infection continuously shapes tumor evolution through chronic immune dysfunction, inflammation, and genomic instability. These processes facilitate the emergence of premalignant clones, accumulation of somatic mutations, and progressive clonal diversification. Under selective pressures imposed by immune surveillance and therapy, resistant tumor subclones emerge and acquire immune-evasive phenotypes. This evolutionary process ultimately culminates in advanced disease characterized by intratumoral heterogeneity, metastatic dissemination, and resistance to conventional and immune-based therapies.

Accumulating evidence suggests that HIV-associated malignancies should be viewed through an evolutionary framework in which chronic immune dysfunction continuously shapes tumor initiation, clonal diversification, immune escape, and therapeutic resistance (Figure 3). The major HIV-driven hallmarks of cancer and the corresponding emerging therapeutic strategies are summarized in Tables 1, 2.

**Table 1:** HIV-driven hallmarks of cancer and their underlying molecular mechanisms.

Hallmark of cancer	HIV-associated mechanisms	Key HIV/viral factors	Major molecular mediators	Representative HIV-associated malignancies	Therapeutic implications
Sustaining proliferative signaling	Persistent activation of oncogenic signaling pathways promotes uncontrolled cellular proliferation	Tat, Nef, EBV LMP1, HHV-8 vGPCR	NF-κB, PI3K–AKT, MAPK, JAK–STAT, MYC	DLBCL, Burkitt lymphoma, Kaposi sarcoma, HCC	PI3K inhibitors, JAK inhibitors, targeted therapies
Evading growth suppressors	Suppression of tumor suppressor pathways and cell-cycle checkpoints	Tat, HHV-8 LANA, HPV E6/E7	p53, RB, E2F	Burkitt lymphoma, Kaposi sarcoma, anal cancer, cervical cancer	Cell-cycle targeted therapy
Resisting cell death	Enhanced anti-apoptotic signaling promotes survival of transformed cells	Nef, EBV, HHV-8	BCL2, Survivin, AKT, NF-κB	HIV-associated lymphomas, Kaposi sarcoma	BCL2 inhibitors, apoptosis-targeting agents
Enabling replicative immortality	Viral latency and immortalization support unlimited proliferation	EBV, HHV-8	LANA, EBNA2, telomerase activation	Primary effusion lymphoma, DLBCL, Kaposi sarcoma	Viral-targeted therapies
Inducing angiogenesis	HIV and viral proteins stimulate vascular remodeling and neovascularization	Tat, HHV-8 vGPCR, vIL-6	VEGF, bFGF, MMPs, IL-6	Kaposi sarcoma, hepatocellular carcinoma	Anti-VEGF therapies
Activating invasion and metastasis	Chronic inflammation and matrix remodeling facilitate dissemination	Tat, TAMs, HIV-induced cytokines	TGF-β, MMPs, CXCL8	Lung cancer, HCC, advanced lymphomas	Anti-metastatic strategies
Genome instability and mutation	DNA damage accumulation and defective repair accelerate oncogenesis	Vpr, chronic inflammation	ATR, DDR pathways, UNG2, ROS	Lung cancer, lymphoma, HCC	DNA damage response inhibitors
Tumor-promoting inflammation	Persistent immune activation establishes a pro-tumorigenic environment	HIV infection, Tat, Vpr	IL-6, TNF-α, IFN-α, CXCL8	Nearly all HIV-associated malignancies	Anti-inflammatory therapies
Avoiding immune destruction	Immune exhaustion and checkpoint activation impair antitumor immunity	HIV, EBV, HHV-8	PD-1, PD-L1, CTLA-4, TIM-3, LAG-3	NHL, KS, HCC, anal cancer	Immune checkpoint blockade
Deregulating cellular energetics	Metabolic reprogramming supports malignant growth and survival	HIV proteins, chronic inflammation	mTOR, HIF-1α, glycolysis pathways	Multiple HIV-associated tumors	Metabolic-targeted therapies
Non-mutational epigenetic reprogramming	Viral and inflammatory signals reshape transcriptional programs	Tat, Vpr, EBV proteins	Histone modification, chromatin remodeling	Lymphoma, KS	Epigenetic therapies
Unlocking phenotypic plasticity	Chronic immune stress promotes cellular adaptation and lineage flexibility	HIV-induced inflammation	EMT pathways, TGF-β signaling	Lung cancer, HCC	Differentiation therapies
Senescent cell effects	HIV-associated senescence generates tumor-promoting SASP factors	Vpr, chronic inflammation	IL-6, IL-1β, SASP pathways	Lung cancer, liver cancer	Senolytic therapies
Polymorphic microbiomes	HIV-associated dysbiosis and microbial translocation enhance systemic inflammation	HIV infection	LPS, microbial metabolites, gut barrier dysfunction	Gastrointestinal cancers, HCC	Microbiome modulation

**Table 2:** Emerging therapeutic strategies in HIV-associated cancer.

Strategy	Mechanistic rationale	Status in HIV+ cancer patients
Immune checkpoint inhibitors (PD-1/PD-L1: nivolumab, pembrolizumab, atezolizumab, durvalumab)	Reverse T-cell exhaustion driven by chronic antigen exposure and HIV-associated immune dysfunction	Comparable ORR/DCR and irAE profile to HIV-negative patients across NSCLC and other HIV-associated cancers; historically excluded from trials (Nageeb, 2025; Alatawi et al., 2025)
CTLA-4 blockade (ipilimumab)	Restores early T-cell priming/co-stimulation	Limited case-series data in HIV+ patients; higher irAE concern given baseline immune activation
Optimized ART regimens	Suppresses viral replication, reduces chronic immune activation, and inflammatory cytokine burden	Standard of care; integrase-inhibitor-based regimens preferred to limit metabolic/cardiovascular toxicity
Anti-herpesvirus/antiviral adjuncts (e.g., targeting KSHV lytic replication)	Limits lytic viral gene products that drive angiogenesis and paracrine oncogenesis	Preclinical/early clinical; no approved KSHV-specific oncolytic strategy
CAR-T/adoptive cell therapy	Redirects cytotoxic activity independent of the exhausted endogenous TCR repertoire	Emerging; feasibility shown in small HIV+ lymphoma cohorts

### 10.1 Application of immune checkpoint inhibitors

ICIs, including antibodies targeting PD-1 and CTLA-4, have revolutionized cancer therapy by reinvigorating exhausted T cells and restoring antitumor immunity. In the context of HIV-associated malignancies, the application of ICIs presents both promising therapeutic potential and unique challenges. HIV-related tumors, such as Kaposi sarcoma (KS) and lymphomas, arise in the setting of chronic immune dysfunction and viral co-infections, notably with HHV-8 and EBV. These tumors often exhibit immune evasion mechanisms that may be susceptible to checkpoint blockade. Preliminary clinical data indicate that ICIs, particularly anti-PD-1 agents like pembrolizumab, can induce objective tumor responses in HIV-positive patients with KS and lymphomas, although response rates are generally lower than those observed in HIV-negative populations (Berhan et al., 2022; Toner et al., 2024). This discrepancy may reflect the complex interplay between HIV-induced immune dysregulation, viral oncogenesis, and tumor microenvironment factors that modulate immune responsiveness. Tumor-associated microbial metabolites may also shape ICI efficacy; microbiota-derived indole-3-lactic acid has been shown to suppress anti-PD-1 responses in esophageal squamous cell carcinoma (Zhou et al., 2026), highlighting a microbiome-immunity axis relevant to precision immunotherapy.

Importantly, the use of ICIs in PLWH necessitates careful consideration of immune-related adverse events (irAEs) and the risk of HIV viral rebound. While ART effectively suppresses HIV replication, immune activation triggered by checkpoint blockade could potentially disrupt viral latency and precipitate viral replication or IRIS. However, emerging evidence suggests that ICIs can be safely administered alongside ART, with manageable toxicity profiles and without significant detrimental effects on HIV control (Silverberg et al., 2021; Parra-Lara et al., 2023). Nonetheless, vigilant monitoring for irAEs and viral load fluctuations remains essential.

Clinical trials evaluating pembrolizumab in HIV-associated KS and lymphomas have demonstrated objective response rates, but these are often modest compared to non-HIV cohorts. For example, pembrolizumab has shown efficacy in inducing tumor regression in KS, a vascular neoplasm driven by HHV-8 infection, which remains prevalent among PLWH despite ART (Ma and Liu, 2022; Lurain et al., 2024). Similarly, ICIs have exhibited activity in HIV-associated non-Hodgkin lymphomas, which are frequently EBV-driven and characterized by profound immunosuppression (Berhan et al., 2022). The lower response rates may be attributable to persistent immune exhaustion, altered T-cell repertoires, and the immunosuppressive tumor microenvironment shaped by chronic HIV infection.

To enhance therapeutic outcomes, combination strategies integrating ART with ICIs are under active investigation. The rationale is to simultaneously suppress HIV replication, restore immune competence, and potentiate antitumor immunity. ART maintains viral suppression and improves CD4+ T-cell counts, which may synergize with checkpoint blockade to overcome tumor-induced immune evasion (Silverberg et al., 2021). Moreover, combining ICIs with conventional chemotherapy or targeted agents may further augment efficacy by modulating the tumor microenvironment and enhancing antigen presentation. However, these approaches require careful balancing to avoid exacerbating immune activation or precipitating toxicities.

Recent systematic evidence reinforces this picture. A systematic review of six cohort studies (*n* = 762) of PD-1 inhibitors (nivolumab, pembrolizumab, atezolizumab, durvalumab) in people with HIV and non-small-cell lung cancer reported a median progression-free survival of 3.0–6.3 months, overall survival of 10.0–66.0 months, objective response rates of 13%−75%, and disease control rates of 47%−62.5%; immune-related adverse events occurred in 25%−75% of patients (6%−20% grade 3–4), with most patients maintaining HIV virologic control (CD4 > 300 cells/μL, undetectable viral load) (Nageeb, 2025). A broader systematic scoping review spanning 107 cases from 19 studies (2011–2024) of ICIs across HIV-associated cancers similarly found a partial response in 27.1%, stable disease in 23.4%, and complete response in 6.5% of cases, with disease progression in 34.6%; hematologic and endocrine adverse events were common but mostly manageable, and HIV viral loads remained stable in most cases (Alatawi et al., 2025). Together, these data support a safety profile for ICIs that is broadly comparable to the general population, while underscoring that disease progression remains a concern and that further controlled trials and optimized patient selection are needed.

### 10.2 Challenges and optimization of CAR-T cell therapy

CAR-T therapy has emerged as a transformative immunotherapeutic approach for treating aggressive B-cell malignancies, including HIV-associated B-cell lymphomas. Despite its promise, the application of CAR-T therapy in HIV-infected individuals faces unique challenges primarily due to chronic immune dysfunction and T-cell exhaustion induced by HIV infection. HIV infection leads to quantitative and qualitative defects in T cells, including diminished proliferative capacity, impaired cytotoxic function, and increased expression of inhibitory receptors, which collectively compromise the efficacy of CAR-T cell manufacturing and persistence after infusion. These immune impairments can reduce the yield and functionality of autologous CAR-T cells, limiting their antitumor activity and durability in HIV-associated lymphomas. Moreover, the chronic antigenic stimulation and immune dysregulation characteristic of HIV infection may exacerbate T-cell exhaustion, further impairing CAR-T cell efficacy and persistence. Consequently, optimizing CAR-T cell therapy for HIV-associated B-cell lymphomas necessitates strategies to overcome these intrinsic T-cell defects and enhance CAR-T cell fitness.

One promising avenue for optimization involves engineering CAR constructs tailored to the HIV-infected milieu. Incorporating HIV-specific CARs that target viral antigens expressed on lymphoma cells or HIV reservoirs can potentially enhance specificity and efficacy. Additionally, the choice of co-stimulatory domains within the CAR construct critically influences T-cell activation, proliferation, and persistence. For instance, the inclusion of the 4-1BB (CD137) co-stimulatory domain has been shown to promote CAR-T cell longevity and reduce exhaustion compared to CD28-based constructs, which may be particularly advantageous in the context of HIV-related immune dysfunction. Such design modifications can enhance antitumor activity while mitigating premature CAR-T cell senescence.

Beyond CAR design, managing the toxicities associated with CAR-T therapy remains a critical concern, especially in immunocompromised HIV-infected patients. Cytokine release syndrome (CRS) and neurotoxicity are the most significant adverse events linked to CAR-T therapy. The dysregulated immune environment in HIV-infected individuals may predispose them to heightened or atypical toxicities, necessitating vigilant monitoring and tailored management protocols. Strategies such as early intervention with anti-cytokine therapies (e.g., tocilizumab for CRS) and corticosteroids for neurotoxicity are essential for mitigating these risks without compromising CAR-T cell efficacy.

Clinical evidence, although limited, supports the feasibility and potential efficacy of CAR-T therapy in HIV-associated refractory B-cell lymphomas. Case reports and small series have documented successful anti-CD19 CAR-T cell therapy in HIV-infected patients with relapsed or refractory lymphoma, demonstrating manageable toxicity profiles and encouraging response rates. However, HIV-infected patients have historically been excluded from pivotal CAR-T clinical trials, limiting robust data on safety and efficacy in this population. Recent advocacy and evolving trial designs are gradually increasing inclusion, which will be critical for defining optimal CAR-T strategies in HIV-associated lymphomas (Hentrich, 2021; Liu Y. et al., 2026; Tang and Nastoupil, 2021; Maillie et al., 2026).

### 10.3 Precision therapy targeting virus-host interactions

Precision therapy targeting virus-host interactions represents a promising frontier in the management of HIV-driven malignancies, particularly those associated with oncogenic viruses such as HHV-8, EBV, and HPV. These viruses, often co-infecting individuals with HIV, significantly contribute to the pathogenesis of both AIDS-defining and non-AIDS-defining cancers by exploiting host cellular machinery and immune dysregulation. Targeted antiviral agents such as ganciclovir analogs (e.g., valganciclovir), mTOR inhibitors like rapamycin, and histone deacetylase inhibitors (HDACi) are currently under clinical investigation for their ability to disrupt these virus-host interactions and impede oncogenesis. For instance, rapamycin has demonstrated efficacy in inhibiting mTOR signaling pathways that are frequently activated in HHV-8-associated Kaposi sarcoma, thereby reducing tumor proliferation and angiogenesis. Similarly, HDAC inhibitors can reactivate latent viral genomes, rendering infected cells susceptible to immune clearance or cytotoxic agents, a strategy being explored in HIV-associated lymphomas and HPV-driven cervical cancers. The integration of tumor genomics into therapeutic decision-making further refines this approach. Molecular profiling has identified key oncogenic pathways such as MYC, BCR, and NF-κB signaling as critical drivers in HIV-associated malignancies. Targeting these pathways with specific inhibitors holds the potential to enhance treatment efficacy. For example, NF-κB pathway inhibitors may counteract the pro-survival signals induced by HIV and co-infecting oncogenic viruses, thereby sensitizing tumor cells to apoptosis. Moreover, the complexity of HIV-driven tumor ecosystems necessitates a multidisciplinary management model that synergizes expertise from infectious disease specialists, oncologists, and immunologists. This collaborative approach ensures comprehensive care encompassing antiretroviral therapy optimization, targeted cancer treatment, and immune modulation. Emerging data underscore the importance of monitoring immune exhaustion markers such as PD-1 and CD160 on CD8+ T cells, which may serve as biomarkers for cancer risk and therapeutic response in people living with HIV. Additionally, novel gene-editing technologies like CRISPR-Cas9 offer future avenues for precise disruption of viral genomes within host cells, potentially eradicating latent reservoirs and preventing oncogenic transformation. Collectively, these strategies embody a precision medicine paradigm that addresses the multifaceted interplay between HIV, oncogenic viruses, and host factors, aiming to improve outcomes in this vulnerable patient population (Rist et al., 2025; Allegra et al., 2020; Olwal et al., 2023; Proulx et al., 2022; Chaudhary et al., 2022; Hassan et al., 2025; Ariumi et al., 2025; Dash et al., 2020).

## 11 Conclusion

The intricate interplay between HIV infection and tumor development represents a complex and evolving field that demands a nuanced understanding from both immunological and oncological perspectives. This review has highlighted the multifaceted nature of the HIV-driven tumor ecosystem, emphasizing chronic immune dysfunction, inflammation-mediated microenvironment remodeling, and direct viral oncogenic mechanisms as central drivers of tumor initiation and progression. From an expert standpoint, it is clear that the tumor microenvironment in HIV-infected individuals is uniquely shaped by immune cell reprogramming—such as M2 polarization of macrophages, T-cell exhaustion, and NK cell dysfunction—and the direct influence of viral proteins like Tat, Nef, and Vpr. These factors collectively facilitate immune evasion and genomic instability, underscoring the complexity of tumor biology in the context of HIV.

Balancing the diverse research perspectives, it is evident that the heterogeneity of tumor evolution in HIV-infected patients—from HIV-associated lymphomas to Kaposi's sarcoma and non-AIDS-defining malignancies—poses significant challenges for diagnosis and treatment. The variability in tumor phenotypes and pathogenetic pathways necessitates precision medicine approaches tailored to individual patient profiles. This heterogeneity also reflects the dynamic nature of the tumor ecosystem, influenced by both viral and host factors, which demands integrative research methodologies to unravel the underlying mechanisms.

ART has undeniably transformed the landscape by restoring immune competence and reducing the incidence of certain HIV-associated malignancies. However, the review underscores that ART alone is insufficient to fully mitigate cancer risk, given the persistent microenvironmental abnormalities and potential drug-drug interactions that may influence tumor behavior and treatment outcomes. This highlights the need for vigilant clinical management and the development of therapeutic regimens that consider the unique pharmacological and immunological milieu of HIV-infected patients.

Emerging therapeutic strategies, including immune checkpoint inhibitors, CAR-T therapies, and targeted interventions aimed at disrupting virus-host interactions, represent promising avenues to enhance anti-tumor efficacy. Nonetheless, these approaches face significant hurdles related to efficacy, safety, and the potential for exacerbating immune dysregulation. The expert consensus suggests that optimizing these novel therapies will require a delicate balance between enhancing anti-tumor immunity and avoiding immune-related adverse events, particularly in the context of chronic HIV infection.

Looking forward, the integration of cutting-edge technologies such as single-cell omics and spatial transcriptomics offers unprecedented opportunities to dissect the dynamic changes within the tumor ecosystem at high resolution. These approaches can illuminate cellular heterogeneity, intercellular communication, and spatial organization, thereby providing critical insights into tumor evolution and immune escape mechanisms. Such knowledge is pivotal for designing rational combination therapies that concurrently target viral persistence and tumor progression.

In conclusion, advancing the management of HIV-associated malignancies hinges on a comprehensive, multidisciplinary approach that reconciles the complexities of viral oncogenesis, immune dysfunction, and tumor heterogeneity. Future research must prioritize the development of integrated therapeutic strategies that combine antiviral and antitumor modalities, tailored to the unique biological context of HIV infection. By fostering collaboration across immunology, virology, oncology, and bioinformatics, the field can move toward improving clinical outcomes and quality of life for people living with HIV who face the added burden of cancer. This balanced and forward-looking perspective is essential to navigate the challenges and harness the opportunities presented by the evolving understanding of the HIV-driven tumor ecosystem. Future advances in multi-omics technologies, biomarker-guided patient stratification, and precision immunotherapy may enable the development of individualized therapeutic strategies for HIV-associated cancers.
